# Electron Microscopic Detection of Single Membrane Proteins by a Specific Chemical Labeling

**DOI:** 10.1016/j.isci.2019.11.025

**Published:** 2019-11-16

**Authors:** Shigekazu Tabata, Marijo Jevtic, Nobutaka Kurashige, Hirokazu Fuchida, Munetsugu Kido, Kazushi Tani, Naoki Zenmyo, Shohei Uchinomiya, Harumi Harada, Makoto Itakura, Itaru Hamachi, Ryuichi Shigemoto, Akio Ojida

**Affiliations:** 1 Graduate School of Pharmaceutical Sciences, Kyushu University, Maidashi, Higashi-ku, Fukuoka, Japan; 2 Institute of Science and Technology Austria, 3400 Klosterneuburg, Austria; 3 Department of Biochemistry, Kitasato University School of Medicine, Sagamihara, Kanagawa, Japan; 4 Department of Synthetic Chemistry and Biological Chemistry, Graduate School of Engineering, Kyoto University, Katsura, Nishikyo-ku, Kyoto, Japan

**Keywords:** Nanoparticles, Biochemistry Methods, Structural Biology

## Abstract

Electron microscopy (EM) is a technology that enables visualization of single proteins at a nanometer resolution. However, current protein analysis by EM mainly relies on immunolabeling with gold-particle-conjugated antibodies, which is compromised by large size of antibody, precluding precise detection of protein location in biological samples. Here, we develop a specific chemical labeling method for EM detection of proteins at single-molecular level. Rational design of α-helical peptide tag and probe structure provided a complementary reaction pair that enabled specific cysteine conjugation of the tag. The developed chemical labeling with gold-nanoparticle-conjugated probe showed significantly higher labeling efficiency and detectability of high-density clusters of tag-fused G protein-coupled receptors in freeze-fracture replicas compared with immunogold labeling. Furthermore, in ultrathin sections, the spatial resolution of the chemical labeling was significantly higher than that of antibody-mediated labeling. These results demonstrate substantial advantages of the chemical labeling approach for single protein visualization by EM.

## Highlights

• Developed peptide tag/probe pair enables specific chemical labeling of proteins
• Chemical labeling visualizes GPCRs on cell surface with 1.4-nm gold particles
• Chemical labeling has higher spatial resolution than immunogold labeling in EM
• Chemical labeling shows a few times higher efficiency than immunogold labeling

## Graphical Abstract

**Figure:**
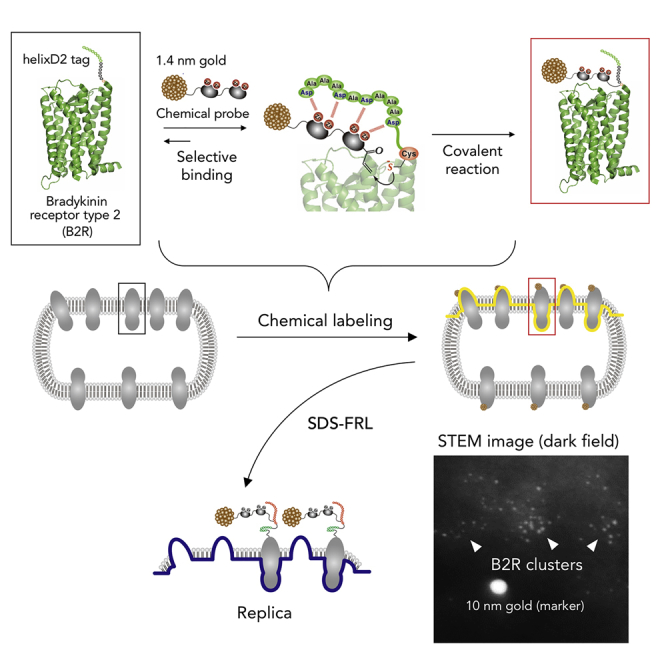

## Introduction

Electron microscopy (EM) is a powerful technique for detecting localization and distribution of a protein of interest (POI) in biological specimens at a nanometer resolution. The most common approach in EM detection of POI is immunogold labeling, in which POI is specifically labeled with a high-electron-density gold particle by using specific antibodies (Faulk and Taylor, 1971). However, immunogold labeling usually suffers from inherent problems, which are mainly associated with the large molecular size of antibody (*ca*. 150 kDa). First, the use of bulky primary and secondary antibodies locates gold particles 20–30 nm away from POI (Fijita et al., 2007), precluding precise detection of protein localization in biological samples. Second, the bulkiness of antibody limits simultaneous labeling of different components of a protein complex. Since numerous proteins function by forming permanent or transient protein complexes in living cells (Srihari et al., 2015, Havugimana et al., 2012), this limitation dramatically diminishes the utility of EM in protein analysis. Third, the large molecular size of antibody hinders its penetration into biological specimens, making it challenging to quantitatively compare POI localization along tissue depth (Masugi-Tokita and Shigemoto, 2007). Fourth, the development of an antibody that is highly selective toward POI is often technically demanding. Chemical protein labeling methods using small molecular probe could potentially overcome these problems. However, despite their extensive use in fluorescence imaging, application to single-protein detection by EM is yet to be reported.

The method for covalent labeling of proteins with a synthetic chemical probe enables functional analyses of POI under biological conditions. Various protein labeling methods have been devised over the past 20 years and mainly used for fluorescence imaging of POI under live cell conditions (Xue et al., 2015, Dean and Palmer, 2014, Krall et al., 2016). Among them, specific cysteine conjugation using reactive peptide tag-probe pair has attracted considerable attention (Schneider and Hackenberger, 2017, Lotze et al., 2016). The pioneering work of Tsien and co-workers led to the development of a method employing a genetically encoded short peptide tag (CysCysXXCysCys) and a biarsenical probe (Griffin et al., 1998). Since then, several peptide tag-based approaches, including oligohistidine tag (His6–10) (Martos-Maldonado et al., 2018, Uchinomiya et al., 2013), π-clamp (Phe-Cys-Pro-Phe) (Zhang et al., 2016), and CX10R7 tag (VTNQECCSIPM) (Ramil et al., 2016), have been devised for specific protein labeling. As a different approach, various enzyme-mediated labeling methods for tag-fused proteins (SNAP-tag, Halo-tag, and lipoic acid ligase) have also been developed (Walper et al., 2017). Compared with these enzymatic approaches, the chemical labeling methods using the tag-probe pair benefit from high tolerability of labeling conditions (including pH, solvent composition, chemical fixation), flexibility in probe design independent from substrate specificity of enzymes, and small molecular size of the tag and the probe, which allows location of the labeled probe at close position to POI. All these properties would increase utility of the chemical labeling method in high-resolution protein analysis by EM, which often needs to handle chemically fixed cell and tissue.

**Figure 1:**
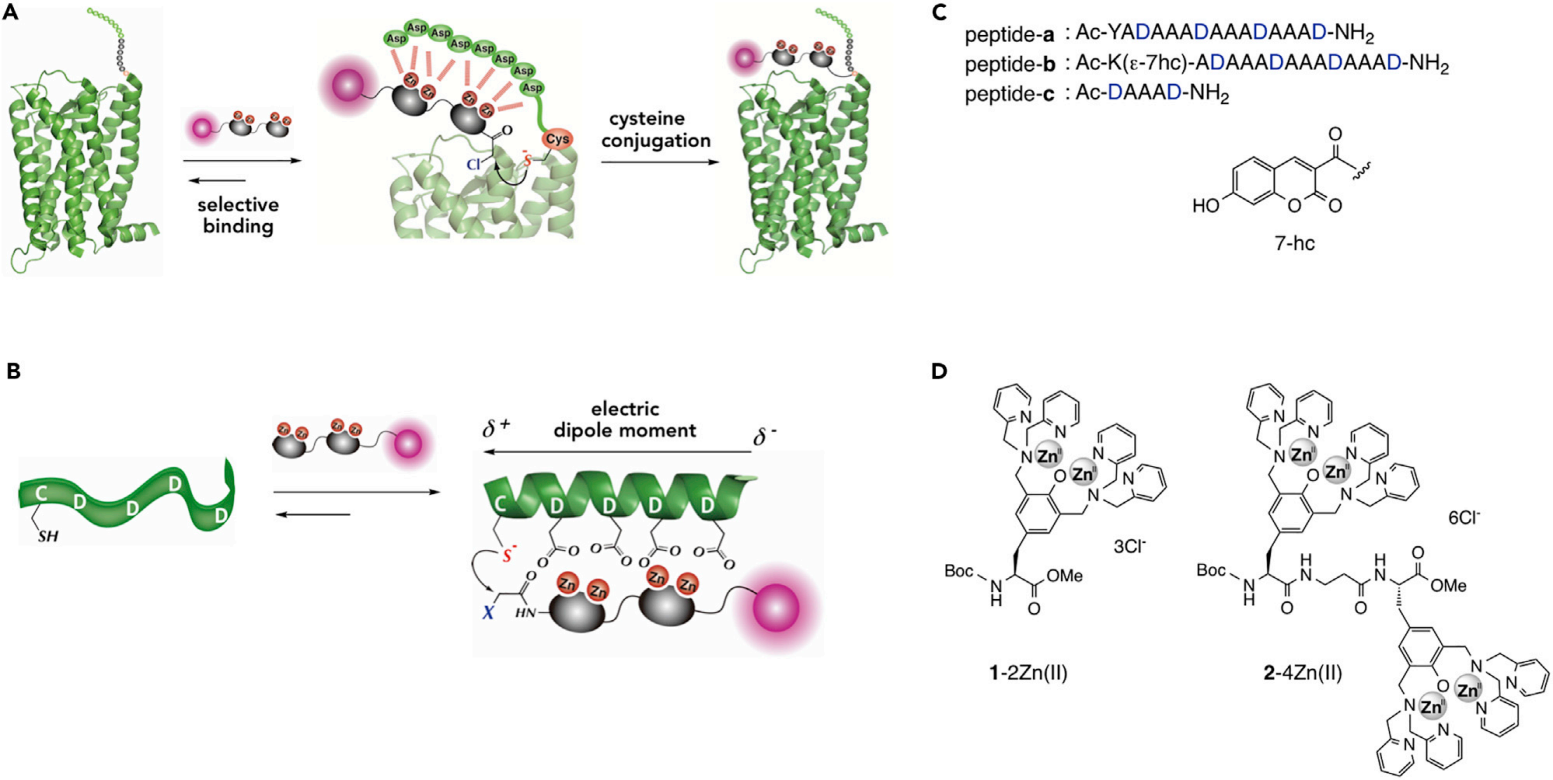
Specific Labeling of Protein Utilizing Complementary Recognition Pair of Peptide Tag and Reactive Zinc Complex (A) Schematic illustration of covalent labeling of tag-fused protein with zinc complex probe. (B) Binding-induced cysteine conjugation of α-helical peptide tag with zinc complex probe developed in the current study. (C) Sequences of the peptide tags. (D) Structures of the Zn(II)-DpaTyr probes.

We have previously reported the selective covalent labeling of protein using a complementary recognition pair of a peptide tag and a reactive zinc complex (Figure 1A) (Nonaka et al., 2010). In this tag-probe pair, the interaction between the aspartate-rich peptide tag (D4-tag; CAAAAAADDDDGDDDD) and the tetranuclear zinc complex (Zn(II)-DpaTyr) induces their cysteine conjugation. However, the overly reactive α-chloroacetamide group of the zinc complex often induced non-specific labeling, hampering its wide use in fluorescence analysis of proteins. This problem prompted us to develop a new tag-probe pair, which has an improved labeling selectivity while retaining a high reactivity. In this manuscript, we report the development of a new peptide tag-probe pair for specific protein labeling and its application to EM detection of membrane proteins at single-molecular level. The high labeling selectivity of this chemical labeling overcame the problem of the non-specific labeling, enabling EM detection of the labeled G protein-coupled receptor (GPCR) in cell membrane freeze-fracture replicas and ultrathin sections. The efficiency and resolution obtained by the chemical labeling was significantly higher than those obtained by the immunogold labeling. The chemical labeling method also revealed high-density clusters of molecules closer than a few nanometers to each other, demonstrating its utility in single protein detection by EM.

## Results and Discussions

### Design of α-Helical Peptide Tag

**Figure 2:**
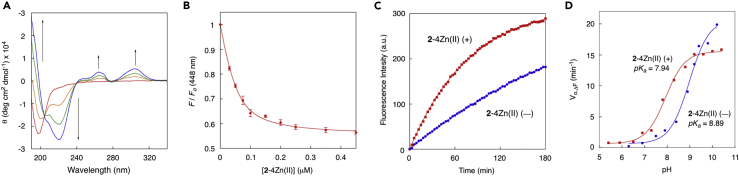
Binding and Reactivity Profiles of Aspartate-Rich Peptide Tags (A) CD spectral change of peptide-**a** upon the addition of **1**-2Zn(II). Conditions: [peptide-**a**] = 50 μM, [**1**-2Zn(II)] = 0, 25, 50, and 100 μM, 10 mM borate buffer, pH 8.0, 25°C. (B) Fluorescence titration profile of peptide-**b** (λ_em_ = 448 nm) upon the addition of **2**-4Zn(II). Conditions: [peptide-**b**] = 0.05 μM, 50 mM HEPES buffer, 100 mM NaCl, pH 7.2, 25°C. Each plot represents the average value ±standard error of triplicate experiments. (C) Time-trace plot of the reaction of peptide-**d** with fluorogenic mCBI in the presence or absence of **2**-4Zn(II). Conditions: [peptide-**d**] = 4 μM, [mCBI] = 120 μM, [**2**-4Zn(II)] = 14 μM, 50 mM HEPES, 100 mM NaCl, pH 7.2, 37°C. (D) Plot of pH-dependent reaction rate (V_o, ΔF_) of peptide-**d** with mCBI in the presence or absence of **2**-4Zn(II). Data were analyzed using Henderson-Hasselbach equation.

The selective chemical labeling of proteins would be achieved by utilizing a pair of highly reactive peptide tag and probe with a tuned weak reactivity. On the basis of this strategy, we initially developed a highly reactive peptide tag with a low *pK*_*a*_ value of the cysteine residue. The *pK*_*a*_ value of a cysteine thiol is typically around 8.5, which indicates that the cysteine residue mainly exists as a weakly reactive sulfhydryl form (R-SH) under neutral physiological conditions (pH = *ca*. 7.4). It is known that an N-terminal cysteine residue of an α-helix possesses a low *pK*_*a*_ value because of the strong electric dipole moment along the helical axis (Figure 1B) (Kortemme and Creighton, 1995). On this basis, we initially designed peptide-**a** with four aspartates at *i*, *i+4* position (Figure 1C). Conformational change of peptide-**a** upon interaction with the binuclear zinc complex **1**-2Zn(II) (Figure 1D) was analyzed by circular dichroism (CD) (Figure 2A). Peptide-**a** existed as a random coil in native state, as indicated by the CD spectrum (10 mM borate buffer, pH 8.0). However, upon the addition of **1**-2Zn(II), the characteristic signals of α-helix were observed at 190, 208, and 222 nm in the CD spectrum, with a concomitant appearance of the induced CD (*i*-CD) peaks at 264 and 303 nm in the absorption region of **1**-2Zn(II). A similar CD signal was also induced by the dimer-type zinc complex **2**-4Zn(II) (Figure S1). The binding affinity of **2**-4Zn(II) to peptide-**a** was estimated to be 1.37 × 10^7^ M^−1^by fluorescence titration of peptide-**b**, bearing a fluorescent 7-hydroxycoumarin (7-hc) (Figure 2B). This binding affinity was approximately 2000-fold higher than that of the binuclear **1**-2Zn(II) and the short peptide-**c** (*K*_*a*_ = 7.04 × 10^3^ M^−1^), as determined by isothermal titration calorimetry measurement (Figure S2). These data suggested that multiple coordination interactions cooperatively work in the binding complex of **2**-4Zn(II) and peptide-**a** to afford the strong binding affinity.

### Design of Peptide Tag with a Highly Reactive Cysteine

We next evaluated the *pK*_*a*_ value of the cysteine residue in the aspartate-rich peptides by using fluorogenic monochlorobimane (mCBI). As shown in Figure 2C, the initial reaction rate of the peptide-**d** with mCBI (V_o,ΔF_, min^−1^) increased with rising pH (Figure 2D), and the analysis of the pH-dependent plot using Henderson-Hasselbalch equation determined its cysteine *pK*_*a*_ value to be 8.89 (Table 1) (Bulaj et al., 1998). Interestingly, this *pK*_*a*_ value decreased to 7.94 in the presence of **2**-4Zn(II), suggesting that the α-helical conformation induced by **2**-4Zn(II) stabilized the thiolate anion due to the helix dipole moment.

**Table 1:** Summary of the *pK*_*a*_ Values of the Cysteine Residue in the Peptides, and the First-Order Reaction Rate Constants (*k*, s^−1^) with mCBI in the Presence or Absence of **2**-4Zn(II)

	2-4Zn(II) (−)		2-4Zn(II) (+)	
	p*K*_*a*_	*k* (× 10^−2^, s^−1^)^a^	p*K*_*a*_	*k* (× 10^−2^, s^−1^)^a^
peptide-**d**: Ac-YG**C**AAADAAADAAADAAAD-NH_2_	8.89	0.66	7.94	1.51
peptide-**e**: Ac-GG**C**PYADAAADAAADAAAD-NH_2_	8.48	2.00	7.46	5.11
peptide-**f**: Ac-YG**C**PAADAAADAAADAAAD-NH_2_	8.93	0.37	7.80	1.24
peptide-**g**: Ac-KK**C**PYSDAAADAAADAAAD-NH_2_	8.04	2.51	7.13	7.60
peptide-**h**: Ac-RR**C**PYSDAAADAAADAAAD-NH_2_	8.02	2.92	7.18	6.72
peptide-**i**: Ac-YG**C**AAAAAADDDDGDDDD-NH_2_	8.96	1.17	8.22	1.78

a Measurement conditions: 50 mM HEPES, 100 mM NaCl, pH 7.2, 37°C.

We then modified the sequence of peptide-**d** to lower the *pK*_*a*_ of the cysteine thiol. It is known that the family of glutaredoxin proteins share a common -Cys-Pro-Xaa-Cys- motif in the active site and that the cysteine thiol positioned at the N terminus of an α-helix has an extremely low *pK*_*a*_ value (*ca*. 3.8) (Jao et al., 2006, Floppe and Nilsson, 2007). On this basis, we designed peptide-**e** (Table 1) with an N-terminal -Cys-Pro-Tyr-Ala- sequence mimicking yeast glutaredoxin. The *pK*_*a*_ of the cysteine thiol of peptide-**e** in the absence of **2**-4Zn(II) was determined to be 8.48, which was lower by approximately 0.4 pH unit than that of peptide-**d** (*pK*_*a*_ = 8.89). Another strategy of lowering the *pK*_*a*_ value of cysteine thiol was the introduction of a positively charged amino acid, which can electrostatically stabilize an adjacent cysteine residue (Lutolf et al., 2001). We thus designed peptide-**g** and peptide-**h**, which possess two Lys or Arg residues at the N termini, respectively. The *pK*_*a*_ values of these peptides (*pK*_*a*_ = 8.04 and 8.02, respectively) were apparently lower than that of peptide-**e** (*pK*_*a*_ = 8.48), indicative of electrostatic stabilization of the thiolate anion by the positively charged amino acid residues. Most interestingly, the *pK*_*a*_ values of all peptides largely decreased by approximately 1 pH unit in the presence of **2**-4Zn(II). The lowest *pK*_*a*_ value (*pK*_*a*_ = 7.13) was observed in the binding complex of peptide-**g** with **2**-4Zn(II). Conversely, the previously developed peptide-**i** (D4-tag) (Nonaka et al., 2010) showed the highest *pK*_*a*_ value (*pK*_*a*_ = 8.22) among the peptides.

The first-order reaction rate constant (*k*, s^−1^) of the peptides with mCBI under neutral aqueous solution (50 mM HEPES, 100 mM NaCl, pH 7.2, 37°C) is summarized in Table 1. Notably, the reaction rate order correlated well with that of the *pK*_*a*_ value of the cysteine thiol. That is, a peptide with a low *pK*_*a*_ exhibited high reactivity, suggesting that the thiolate anion served as the main reactive species in the nucleophilic reaction with mCBI. Furthermore, **2**-4Zn(II) accelerated the reaction of all α-helical peptides (peptides-**d**–**h**) with mCBI, consistent with their low *pK*_*a*_ values in the binding complex. The most reactive peptide was peptide-**g**: its reaction rate in the presence of **2**-4Zn(II) (*k* = 7.60 × 10^−2^ s^−1^) was over ten times higher than that of the initially designed peptide-**d** in the absence of **2**-4Zn(II) (*k* = 0.66 × 10^−2^ s^−1^). These observations demonstrated the validity of our strategy to gain a highly reactive peptide tag by lowering the *pK*_*a*_ value of the cysteine.

### Tuning of Probe Reactivity for Enhanced Labeling Selectivity

To reduce the non-specific labeling activity of the Zn(II) complex, we next tuned its reactivity. Michael acceptor is an important class of reactive group for cysteine thiol and has been widely used for protein modification with synthetic probes and covalent drugs (Singh et al., 2010). Considering the broad tunability of its reactivity (Flanagan et al., 2014), we prepared the monomer-type zinc complexes **3**-2Zn(II) to **5**-2Zn(II) bearing a different Michael acceptor group (Figure S3) and evaluated their reactivity with peptide-**g** by using mCBI. The kinetic analysis revealed that the reactivity of **5**-2Zn(II) (*t*_*1/2*_ = 3.98 h) bearing a γ-dimethylaminocrotonate (DMAC) (Wissner et al., 2003) as Michael acceptor group was substantially lower than that of α-chloroacetamide **6**-2Zn(II) (*t*_*1/2*_ = 0.20 h). These data suggested the potential utility of DMAC as a reactive group of the labeling probe with a moderate reactivity. The other Michael acceptor-type probes 3-2Zn(II) and 4-2Zn(II), which bears a cinnamide and crotonamide, respectively, showed the low reactivity with peptide-**g** (*t*_*1/2*_ > 24 h).

We next evaluated the reactivity of dimer-type zinc complexes **7**-4Zn(II) to **9**-4Zn(II) bearing a DMAC group (Table 2). Their reactivity was evaluated with peptide-**g** to peptide-**i**, which possessed the different number of alanine residue(s) for optimization of spatial orientation between the cysteine residue and the bound Zn(II) complex. Interestingly, the reactivity was greatly affected by the structure of the Zn(II) complex: the reaction rate of **9**-4Zn(II), possessing a rigid L-proline linker, with peptide-**g** (*t*_*1/2*_ = 0.74 h) was higher than that of **7**-4Zn(II) (*t*_*1/2*_ = 3.3 h). In contrast, **8**-4Zn(II) possessing an ethylene glycol linker exhibited a much lower reactivity (*t*_*1/2*_ = 13.9 h). The reactivity was also influenced by the number of inserted alanine(s) in the peptide tag: peptide-**k**, which possessed two additional alanine residues, showed a higher reactivity (*t*_*1/2*_ = 0.22 h) for **9**-4Zn(II) compared with peptide-**g** (*t*_*1/2*_ = 0.74 h). Consequently, **9**-4Zn(II) and peptide-**k** were identified as the optimized tag-probe pair with the highest reactivity. We confirmed that the *pK*_*a*_ values of peptides with inserted alanine(s) (peptide-**j** and -**k**) in a binding complex with **2**-4Zn(II) were 7.08 and 7.14, respectively, which were comparable with that of peptide-**g** (*pK*_*a*_ = 7.13) (Figure S4). Overall, the reactivity of the optimized tag-probe pair (i.e., **9**-4Zn(II) and peptide-**k**, *t*_*1/2*_ = 0.22 h) was approximately 30-fold higher than that of the previously developed D4-tag peptide-**i** with the structurally unoptimized **7**-4Zn(II) (*t*_*1/2*_ = 6.78 h, Table 2).

**Table 2:** Summary of the Reaction Half-Time (*t*_*1/2*_, h) of the Zinc Complexes with the Peptides


	*t*_*1/2*_ (h)		
	7-4Zn(II)	8-4Zn(II)	9-4Zn(II)
peptide-**g**: Ac-KK**C**PYSDAAADAAADAAAD-NH_2_	3.30	13.9	0.74
peptide-**j**: Ac-KK**C**PYSADAAADAAADAAAD-NH_2_	2.69	16.0	0.24
peptide-**k**: Ac-KK**C**PYSAADAAADAAADAAAD-NH_2_	1.96	9.84	0.22
peptide-**i**: Ac-YG**C**AAAAAADDDDGDDDD-NH_2_	6.78	–a	2.84

Measurement conditions: 50 mM HEPES, 100 mM NaCl, pH 7.2, 37°C.

a Not determined.

### Fluorescence Labeling of HelixD2-Tag-Fused Protein

**Figure 3:**
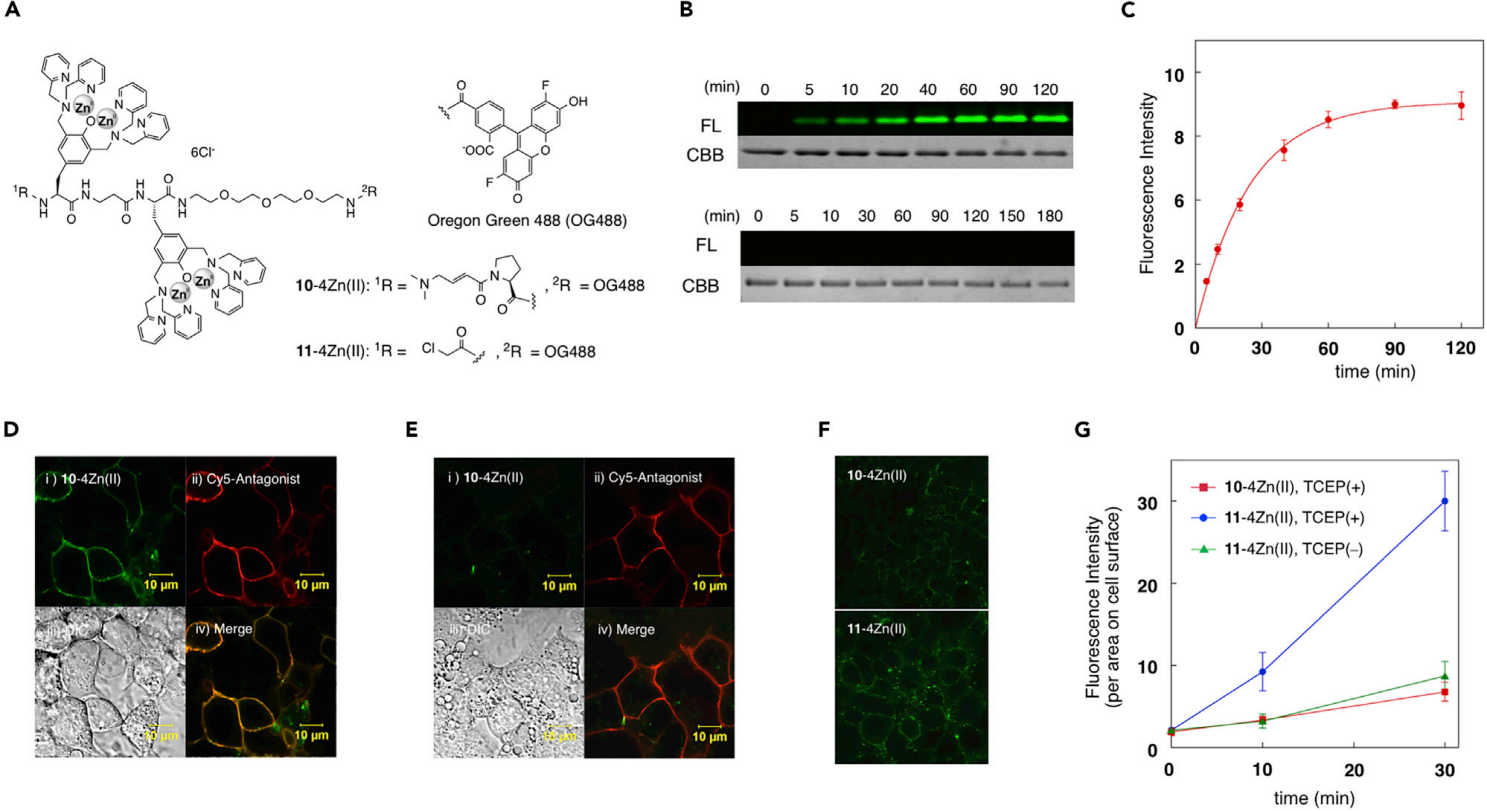
Fluorescence Labeling of helixD2-Tag-Fused Protein (A) Structures of the fluorescent Zn(II)-DpaTyr probes. (B) In-gel fluorescence (FL) and Coomassie Brilliant Blue (CBB) analysis of covalent labeling of MBP tagged with a helixD2-tag (upper panel) or CH6 tag (lower panel) by **10**-4Zn(II). Labeling conditions: [tag-MBP] = 1 μM, [**10**-4Zn(II)] = 10 μM, 50 mM HEPES, 100 mM NaCl, 20 mM TCEP, pH 7.2, 37°C. (C) Time-trace plot of the fluorescence band intensity of the MBP labeled with **10**-4Zn(II) (mean ± SD of triplicate experiment). (D and E) Fluorescence imaging of HEK293 cells expressing B2R fused with helixD2-tag (D) or CH6 tag (E) upon labeling with **10**-4Zn(II). Labeling conditions: [**10**-4Zn(II)] = 4 μM, HEPES-buffered saline, pH 7.4, 37°C, 30 min. (F and G) (F) Fluorescence images of non-specific labeling on surface of HEK293 cells without expression of the tag-fused B2R. Labeling conditions: [**10**-4Zn(II) or **11**-4Zn(II)] = 4 μM, HEPES-buffered saline, 20 μM TCEP, pH 7.4, 37°C, 30 min (G) Time-trace plot of the fluorescence intensity on the surface of HEK293 cells without expression of the tag-fused B2R (n = 10, mean ± SD).

The optimized tag-probe pair was used for fluorescence labeling of tag-fused proteins. We first employed maltose-binding protein (MBP) tagged with the 21-amino acid peptide-**k**, named helixD2-tag (KKCPYSAADAAADAAADAAAD). The labeling reaction of helixD2-MBP was conducted with the fluorescent zinc complex **10**-4Zn(II) bearing an Oregon green 488 under aqueous buffer conditions (50 mM HEPES, 100 mM NaCl, pH 7.2) (Figure 3A). In-gel fluorescent analysis revealed that the labeling reaction proceeded in a time-dependent manner (Figures 3B and 3C). The half reaction time (*t*_*1/2*_) was determined to be 0.28 h by pseudo-first-order kinetic analysis, which was comparable with that of the reaction between peptide-**k** and **9**-4Zn(II) (*t*_*1/2*_ = 0.22 h, Table 2). The second-order reaction rate constant of the labeling reaction of helixD2-MBP with **9**-4Zn(II) was also determined to be 9.4 × 10^2^ M^−1^ s^−1^ by the detailed kinetic analysis (Figure S5). A control labeling reaction was conducted using MBP tagged with the cysteine-containing histidine-rich peptide (CH6 tag; CHHHHHH). As shown in Figure 3B, no labeling adduct was detectable with CH6-MBP, consistent with the weak binding affinity of the zinc complex and hexahistidine peptide (K_a_ < 10^3^ M^−1^) (Uchinomiya et al., 2014).

We next applied the tag-probe pair to the covalent labeling of GPCR protein on the cell surface. In the experiment, bradykinin receptor type2 (B2R) was tagged with a helixD2-tag (located at the extracellular N-terminal region of B2R) and transiently expressed in HEK293 cells. When the cells were pre-treated with tris(2-carboxyethyl)phosphine (TCEP) to activate the cysteine residue of the tag, and then incubated with 4 μM of **10**-4Zn(II) for 30 min at 37°C, the bright fluorescence signal was observed on the cell surface (Figure 3D). The obtained fluorescence image overlapped well with that of the fluorescent B2R antagonist peptide, indicative of selective covalent labeling of the tag-fused B2R by **10**-4Zn(II). The fluorescence of **10**-4Zn(II) was not observed on the cell surface when the labeling reaction was conducted in the presence of 1 mM inorganic pyrophosphate (PPi), a strong binder to the zinc complex (Figure S6). Similarly, the labeling of HEK293 cells expressing B2R tagged with CH6 did not show any fluorescence on the cell surface (Figure 3E). These data suggest that the selective binding between the helixD2-tag and **10**-4Zn(II) is essential for the efficient covalent labeling of B2R.

To evaluate the non-specific labeling activity of the probes on the cell surface, HEK293 cells that did not express B2R were treated with **10**-4Zn(II) or α-chloroacetamide probe **11**-4Zn(II) (4 μM, 37°C), and the fluorescence of the cell surface was quantified by confocal microscopy with a high laser power after formaldehyde fixation (Figures 3F and 3G). The result revealed that the fluorescence of **10**-4Zn(II) associated with non-specific reaction was apparently weaker than that of the highly reactive **11**-4Zn(II). Furthermore, **11**-4Zn(II) induced a noticeable time-dependent increase of the fluorescence signal on the cell surface. These data clearly indicated that non-specific labeling activity of **10**-4Zn(II) was significantly lower than that of **11**-4Zn(II). The fluorescence of **11**-4Zn(II) substantially decreased without the reductive treatment with TCEP, implying that cysteine residue of membrane proteins was the major non-specific labeling site. The weak non-specific reactivity of **10**-4Zn(II) was also confirmed by the labeling experiment of the helixD2-MBP under E. *coli* lysate conditions (Figure S7). In-gel fluorescence analysis of the lysate sample revealed that the band intensities of proteins non-specifically labeled by **10**-4Zn(II) were significantly lower than those labelled by **11**-4Zn(II).

### EM Detection of Tag-Fused Protein Using Biotin and Nanogold-Conjugated Probes in Freeze-Fracture Replica

**Figure 4:**
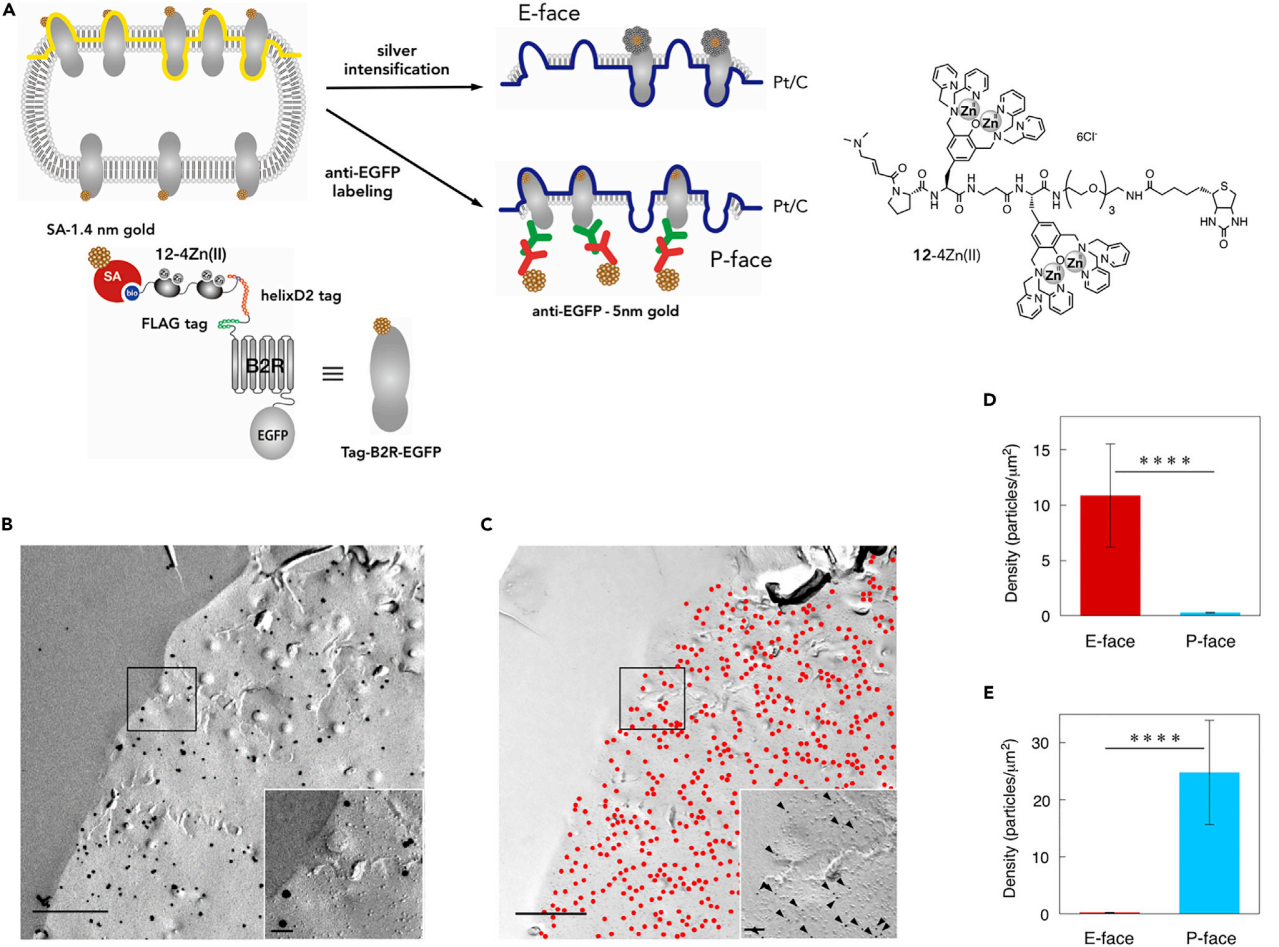
EM Detection of helixD2-Tag-Fused B2R Protein on Freeze-Fracture Replicas (A) Schematic illustration of labeling states of a complementary replica pair. (B and C) EM images of the complementary replicas showing E-face (B) and P-face (C) from the same membrane. Scale bars, 1 μm. (Insets) Magnified images of the area framed in the main pictures. Arrowheads indicate 5-nm gold particles. Scale bars, 100 nm. (D) Comparison of the labeling density of **12**-4Zn(II) on the E- and P-face images, respectively (****p < 0.001, Mann-Whitney U test; E-face, n = 11; P-face, n = 9, mean ± SE). (E) Comparison of the labeling density of anti-GFP antibody on the E- and P-face images, respectively (****p < 0.001, Mann-Whitney U test; E-face, n = 7; P-face, n = 15, mean ± SE).

We applied chemical labeling to EM detection in combination with sodium dodecyl sulfate (SDS)-digested freeze-fracture replica labeling (SDS-FRL) technique (Fujimoto, 1995). This technique is widely used to detect the two-dimensional distribution of membrane proteins on protoplasmic (P-) and exoplasmic (E−) leaflet of lipid bilayer in freeze-fracture replicas (Meier and Beckmann, 2018). In the current study, we first used the biotin probe **12**-4Zn(II) to detect helixD2-B2R-EGFP, which harbored an extracellular N-terminal helixD2-tag and an intracellular C-terminal enhanced green fluorescent protein (EGFP) (Figure 4A). The labeling specificity of **12**-4Zn(II) toward helixD2-B2R-EGFP was confirmed by fluorescence imaging using Alexa Fluoro 633-conjugated streptavidin (SA) (Figure S8). For EM detection, helixD2-B2R-EGFP, expressed on the surface of live HEK293 cells, was labeled with **12**-4Zn(II) followed by secondary labeling with SA-conjugated 1.4-nm gold particle. After fixation with paraformaldehyde, a complementary pair of P- and E-face freeze-fracture replica was prepared using the double replica method (Figure 4A). One replica of the pair underwent silver enhancement to increase the visibility of 1.4-nm gold particles labeled by the chemical labeling on the E-face. In another replica of the pair, EGFP was immunolabeled with 5-nm gold particles to detect helixD2-B2R-EGFP on the P-face. EM analysis revealed selective labeling of the helixD2-tag by the probe on the E-face (Figures 4B and 4D) and vice versa, selective labeling of EGFP on the corresponding P-face (Figures 4C and 4E). These observations are consistent with the extracellular location of the helixD2-tag and the intracellular location of EGFP.

**Figure 5:**
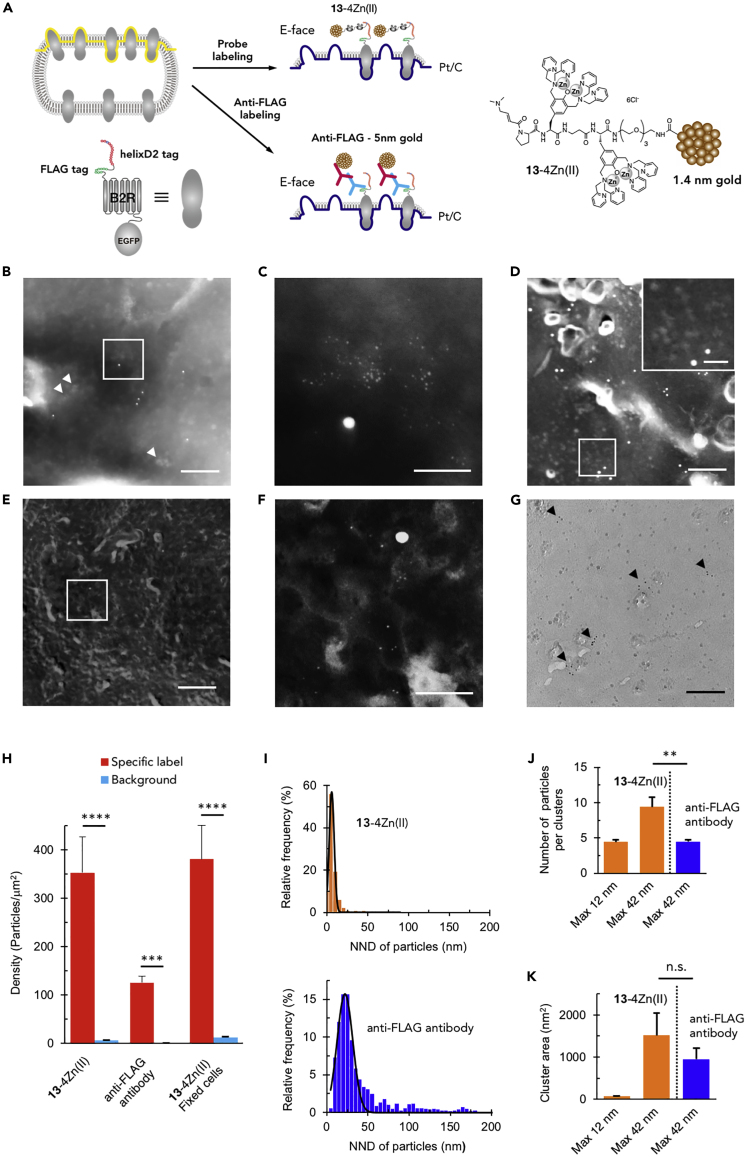
EM Detection of helixD2-Tag-Fused B2R Protein on HEK Cells Using the Probe Conjugated with a 1.4-nm Gold Particle (A) Schematic illustration of labeling states of replicas. (B–D) Dark-field scanning transmission electron microscopy images of E-face replicas made from transfected (B and C) or non-transfected (D) HEK cells labeled with **13**-4Zn(II) before aldehyde fixation. (C) and the inset on (D) are magnified images of the area framed in (B) and (D), respectively. White arrowheads indicate B2R clusters labeled with **13**-4Zn(II). Scale bar: 200 nm in (B) and the main picture of (D) and 50 nm in (C) and the inset on (D). (E and F) Dark-field images of replicas made from transfected HEK cells labeled with **13**-4Zn(II) after aldehyde fixation. (F) A magnified image of the area framed in (E). Scale bar: 200 nm in (E) and 50 nm in (F). (G) Bright-field TEM image of E-face replica labeled with anti-FLAG antibody combined with 5-nm gold-particle-conjugated secondary antibody. Black arrowheads indicate B2R clusters labeled with 5-nm gold particles. Scale bar, 200 nm. (H) Comparison of the specific and background labeling density with **13**-4Zn(II) and anti-FLAG antibody. “**13**-4Zn(II)” and “**13**-4Zn(II), fixed cells” indicate results from HEK cells labeled before and after aldehyde fixation, respectively (**13**-4Zn(II), ****p < 0.001, Mann-Whitney U test; specific labeling, n = 9 cells; background labeling, n = 13 cells; mean ± SE, anti-FLAG, ***p < 0.005, Mann-Whitney U test; specific labeling, n = 7 cells; background labeling, n = 7 cells; mean ± SE, **13**-4Zn(II), fixed cells, ****p < 0.001, Mann-Whitney U test; specific labeling, n = 11 cells; background labeling, n = 15 cells; mean ± SE). (I) Distribution histograms of nearest neighbor distance (NND) of gold particles observed on replicas labeled with **13**-Zn(II) (upper, n = 1748 particles) and anti-FLAG antibody (bottom, n = 998 particles). Fitted lines show Gaussian distribution fitting for the peaks. (J) Comparison of the number of particles per B2R cluster labeled with **13**-4Zn(II) and anti-FLAG antibody. “Max 12 nm” and “Max 42 nm” mean the maximum NND of gold particles used for the definition of clusters labeled with **13**-4Zn(II) and anti-FLAG antibody (**13**-4Zn(II) Max 12 nm; n = 10 cells, **13**-4Zn(II) Max 42 nm, n = 10 cells; anti-FLAG Max 42 nm, n = 7 cells, mean ± SE, **p < 0.01, Mann-Whitney U test). (K) Comparison of the B2R cluster area evaluated by chemical labeling with **13**-4Zn(II) and immunolabeling with anti-FLAG antibody (**13**-4Zn(II) Max 12 nm; n = 10 cells; **13**-4Zn(II) Max 42 nm, n = 10 cells; anti-FLAG 42 nm, n = 7 cells; mean ± SE, n.s. p > 0.05, Mann-Whitney U test).

Although the density of gold particles looked higher for EGFP than helixD2-tag, it is necessary to visualize each of them on the E-face to compare the labeling efficiency, because the allocation of helixD2-B2R-EGFP to the P-face and E-face can be different. In addition, binding of the biotin probe **12**-4Zn(II) and SA-conjugated particle can be disrupted by SDS treatment at 60°C reducing the labeling efficiency. Thus, we next used **13**-4Zn(II), which possesses a directly conjugated 1.4-nm nanogold particle to label helixD2-B2R-EGFP, and anti-FLAG antibody to label FLAG tag, which was inserted just next to the helixD2 tag in the extracellular domain of B2R (Figure 5A). Probe **13**-4Zn(II) was obtained by conjugation reaction of the amine probe **14**-4Zn(II) with mono-sulfo-N-hydroxy-succinimide nanogold particles in HEPES buffer (pH 8.0), which was followed by dialysis to remove the excess of **14**-4Zn(II). The labeling reaction of **13**-4Zn(II) was conducted for an extended time (2 h, 37°C) to fully label helixD2-B2R-EGFP expressed on HEK293 cells. To facilitate searching for E-face labeled with 1.4-nm gold particles by 200kV scanning transmission electron microscope (dark field mode), GluA2 subunit of α-amino-3-hydroxy-5-methyl-4-isoxazolepropionic acid-type glutamate receptor was co-expressed with helixD2-B2R-EGFP in HEK cells and labeled with 10-nm gold particles by an antibody for an extracellular epitope of GluA2 combined with anti-rabbit 10-nm gold particle-conjugated secondary antibody (Figure S9). On GluA2-labeled E-face, we found numerous 1.4-nm gold particles, often making high-density clusters of particles (Figures 5B and 5C). The specificity of the labeling was confirmed using HEK cells transfected with GluA2 but not with helixD2-B2R-EGFP (Figure 5D). The density of background labeling (5.9 particles/μm^2^) was 1.7% of the specific labeling (353 particles/μm^2^) (Figure 5H). In the immunogold labeling, the specific labeling by anti-FLAG tag antibody was observed on the E-face with 5-nm particles (125 particles/μm^2^, n = 7 cells, Figure 5H), also showing clusters of particles (arrowheads in Figure 5G). The average density of B2R labeled with **13**-4Zn(II) was 2.8 times higher than that with anti-FLAG antibody (Figure 5H, p < 0.05).

To compare the clusters of particles obtained with the chemical labeling and anti-FLAG immunolabeling, we first examined nearest neighbor distance (NND) distributions of particles and found sharper peak at smaller NND for the chemical labeling (8.6 ± 10.8 nm, mean ± SD, n = 1748 particles) than immunolabeling (47.4 ± 56.1 nm, mean ± SD, n = 998 particles, p < 0.001, Mann-Whitney U test) (Figure 5I). For defining the clusters, we fitted these peaks with Gaussian distribution and used mean + 2 SD as maximum distances allowed and three particles per cluster as a minimum number of particles per cluster (Miki et al., 2017). In case we use definition of clusters with different mean + 2 SD values for chemical labeling (12 nm) and immunolabeling (42 nm), the numbers of particles per cluster were similar (Figure 5J) but the cluster area was much smaller for chemical labeling (70 nm^2^) than for immunolabeling (950 nm^2^, Figure 5K), indicating that the clusters detected by chemical labeling represent subclusters consisting single clusters detected by immunolabeling. In case we use the same definition of clusters obtained by immunolabeling for both (42 nm as a maximum distance allowed), the cluster areas became similar and the number of particles per cluster was twice higher for chemical labeling than for immunolabeling (9.4 ± 1.4 particles, n = 10 cells for chemical labeling, 4.5 ± 0.3 particles, n = 7 cells for immunolabeling, mean ± SE, p < 0.01, Mann-Whitney U test, Figures 5J and 5K). Altogether, these results indicate higher labeling efficiency and detectability of high-density nanoclusters of proteins for chemical labeling with the probe directly conjugated with 1.4-nm nanogold particle than the immunolabeling with 5-nm gold-particle-conjugated secondary antibody.

In typical SDS-FRL experiment, biological samples, such as sliced tissue, are initially fixed with aldehyde to maintain protein location by physical stabilization (Masugi-Tokita and Shigemoto, 2007). Therefore, we next applied the chemical labeling to the fixed cell samples. HEK293 cells expressing helixD2-B2R-EGFP were first fixed with 2% paraformaldehyde and then labeled with **13**-4Zn(II). We found specific labeling for helixD2-B2R-EGFP with the fixed cells comparable with that obtained with living cells (Figures 5E, 5F, and 5H). This was indicative of the compatibility of the developed chemical labeling with aldehyde fixation. Since the majority of the existing enzyme-mediated and ligand-directed protein labeling methods (Walper et al., 2017, Matsuo and Hamachi, 2017) are difficult to apply to denatured proteins by fixation, these results demonstrated the high utility of the chemical labeling method for SDS-FRL using fixed biological samples.

### EM Detection of Tag-Fused Protein Using Nanogold-Conjugated Probes in Ultrathin Section

**Figure 6:**
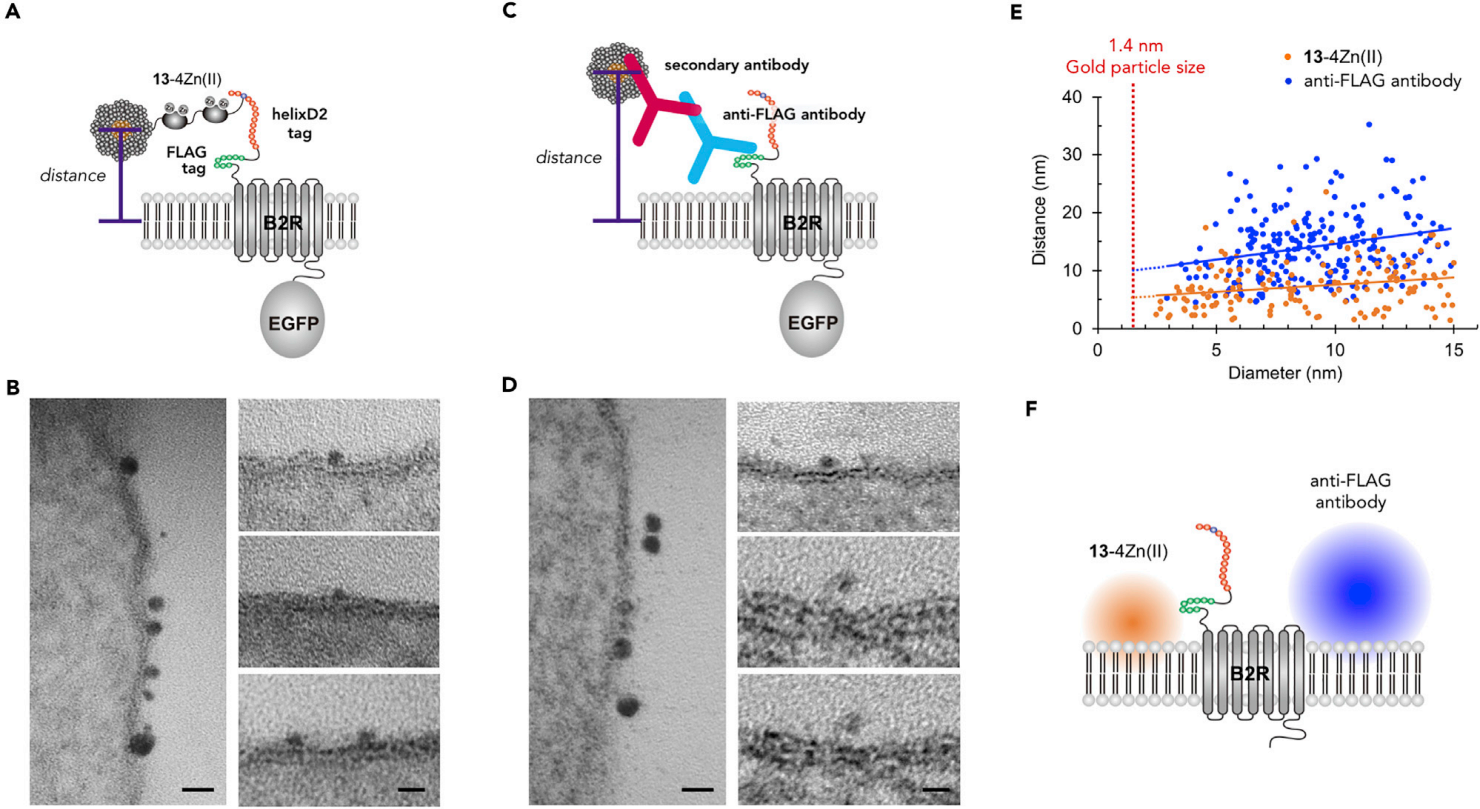
Comparison of Resolution between Chemical Labeling and Immunolabeling Methods (A and C) Schematic illustration of labeling states of the tagged-B2R on HEK cells labeled with **13**-4Zn(II) (A) and anti-FLAG antibody combined with 1.4-nm gold-particle-conjugated secondary antibody (C). (B and D) EM images of ultrathin sections prepared from HEK cells labeled with **13**-4Zn(II) (B) and anti-FLAG antibody (D), respectively. Gold particles were intensified by silver enhancement for 3–6 min. Distances between the particle center and the middle of the cell membrane were measured in sections tilted to obtain perpendicular views to the plasma membrane. Scale bars, 20 nm (left) and 10 nm (right). (E) Correlation of the intensified particle size and distance between particle centers and the middle of the cell membrane (orange dots, **13**-4Zn(II); blue dots, anti-FLAG antibody). Significant positive correlation was detected for both (correlation coefficient = 0.24, p < 0.01, n = 147 for **13**-4Zn(II); correlation coefficient = 0.26, p < 0.01, n = 228 for anti-FLAG antibody, Pearson correlation analysis). The distances for non-intensified 1.4-nm particles were deduced from linear fits extrapolated (broken orange and blue lines) to 1.4 nm (red dotted line). (F) Schematic illustration of positions of non-intensified 1.4-nm particles estimated by the two labeling methods in (E). The gradient circle indicates the deviation of the position.

To further confirm higher resolution of the chemical labeling using **13**-4Zn(II), we performed EM analysis of ultrathin sections of resin-embedded HEK293 cells. We compared the distances from the silver-intensified 1.4-nm gold particles to cell membrane between the samples labeled by **13**-4Zn(II) and those labeled by anti-FLAG antibody combined with 1.4-nm gold-particle-conjugated secondary antibody (Figures 6A and 6C). The background density of particles examined in non-transfected cells was 0.8% and 0.6% of the specific labeling by **13**-4Zn(II) and anti-FLAG antibody, respectively. The silver-intensified gold particles appeared to be mostly attached to the cell membrane in the chemical labeling (Figure 6B), whereas those in the FLAG immunolabeling showed apparent gaps between the particles and the cell membrane (Figure 6D). Considering that silver intensification may occur in a non-isotropic/concentric manner around 1.4-nm gold particles, we used minimum time (3–6 min) for silver intensification and examined the correlation of the size of silver-intensified particles and the distance between the center of silver-intensified particles and the midpoint of lipid bilayer (Figure 6E). We found a significant positive correlation for both chemical labeling (r_s_ = 0.24, p < 0.01, n = 147 particles) and immunolabeling (r_s_ = 0.26, p < 0.01, n = 228 particles). By extrapolating linear fits, estimated distances of non-intensified 1.4-nm particles were calculated to be 5.4 nm and 9.9 nm for chemical labeling and immunolabeling, respectively. Since the half thickness of lipid bilayer was estimated to be 4 nm by EM analysis (Robertson, 1958), the distance of 1.4-nm gold particles from the cell surface was deduced to be 1.4 and 5.9 nm for the chemical labeling and immunolabeling, respectively (Figure 6E). Although direct comparison of the distances from these labels to the respective tags is not possible because the exact tag positions are unknown, the variance of the measured distances is significantly smaller for **13**-4Zn(II) than for anti-FLAG antibody (F_227, 146_ = 2.0, p < 0.001), indicating higher resolution of the chemical labeling method than the immunolabeling method (Figure 6F).

### Conclusion

In summary, we have achieved EM detection of GPCR by chemical labeling using a reactive peptide tag-probe pair. Rational design of the highly reactive α-helical peptide tag and the fine-tuning of probe reactivity enabled the specific cysteine conjugation of the tag-fused B2R protein on cell surface. EM detection using the tag-probe pair was successfully applied to determine the localization of B2R receptor on cell surface with a high labeling specificity. Furthermore, the probe directly conjugated with a 1.4-nm gold particle enabled the detection of the membrane proteins with a few times higher labeling efficiency and significantly higher spatial resolution than that of the antibody-mediated labeling, revealing high-density nanoclusters of proteins. EM detection methods for in-cell proteins using genetically encoded peroxidases have been developed in recent years (Shu et al., 2011, Martell et al., 2012, Hainfeld and Powell, 2000). Despite their usefulness, these methods are hardly applicable to single-protein detection since they have been devised for EM contrast imaging based on OsO_4_ staining in combination with oxidative diaminobenzidine (DAB) polymerization. To the best of our knowledge, the research presented herein is the first example of high-resolution single-protein detection by EM utilizing a chemical labeling. The use of our labeling method is currently limited to cell surface proteins. Nevertheless, this method can be widely used for EM analysis of membrane proteins on replicas in combination with SDS-FRL. We envision further application of the chemical labeling approach to reveal subunit composition of single-protein complexes on cell surface in combination with other tag-probe pairs (Uchinomiya et al., 2013).

### Limitations of the Study

In our study, we demonstrate higher efficiency and resolution of the newly developed chemical labeling method compared with the common immunolabeling methods at the EM level using HEK cells expressing tagged B2R. Accurate evaluation of labeling resolution, however, requires understanding of the exact molecular structure of the tagged receptor. Since background and labeling conditions can be different between cell culture and various tissue environments, usefulness of the reported reactive peptide tag-probe pair needs further verification in more complex sample preparations.

## Methods

All methods can be found in the accompanying Transparent Methods supplemental file.

## Supplemental Information

Document S1. Transparent Methods and Figures S1–S9
