# Supplementary material for: Electron Microscopic Detection of Single Membrane Proteins by a Specific Chemical Labeling
Source: iScience. 2019 Nov 16;22:256–68. doi: 10.1016/j.isci.2019.11.025 (PMC6906691; doi:10.1016/j.isci.2019.11.025)
Supplement: Document S1. Transparent Methods and Figures S1–S9 [file mmc1.pdf]

ISCI, Volume *22*

Supplemental Information

Electron Microscopic Detection of Single Membrane Proteins by a Specific Chemical Labeling

Shigekazu Tabata, Marijo Jevtic, Nobutaka Kurashige, Hirokazu Fuchida, Munetsugu Kido, Kazushi Tani, Naoki Zenmyo, Shohei Uchinomiya, Harumi Harada, Makoto Itakura, Itaru Hamachi, Ryuichi Shigemoto, and Akio Ojida

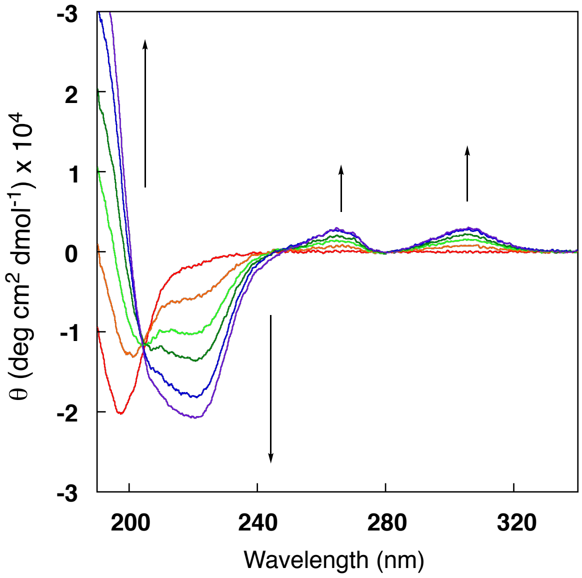

Figure S1. CD spectral change of peptide-**a** upon the addition of **2**-4Zn(II), Related to Figure 2. Measurement conditions: [peptide-**a**] = 50 μM, [**2**-4Zn(II)] = 0, 10, 20, 30, 40, and 50 μM, 10 mM borate buffer, pH 8.0, 25 °C.

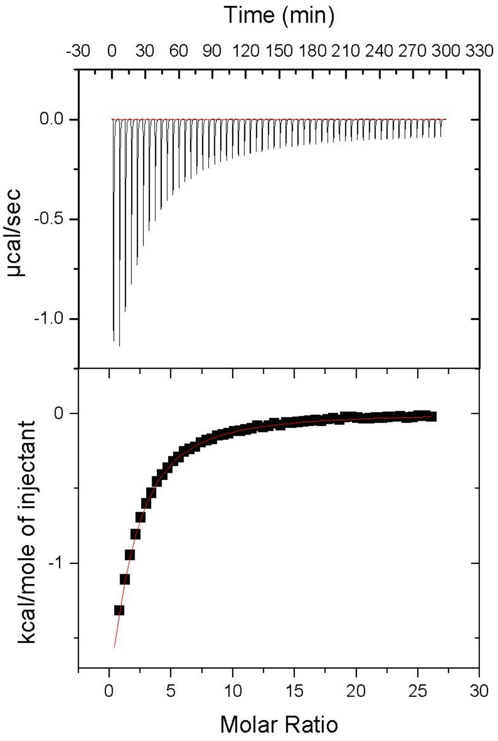

n	0.97 ± 0.06
K_{app} (M^{-1})	7.04 ± 0.15 x 10^3
ΔH (kcal/mol)	-6.67 ± 0.46
TΔS (kcal/mol)	-1.41

Figure S2. ITC titration curve (upper) and processed data (lower) of **1**-2Zn(II) with peptide-**c** (Ac-DAAAD-NH_2), Related to Figure 2. [**1**-2Zn(II)] = 50 μM, [peptide-**c**] = 6 mM (5 μL × 48 injections), 50 mM HEPES, 100 mM NaCl, pH 7.2, 25°C.

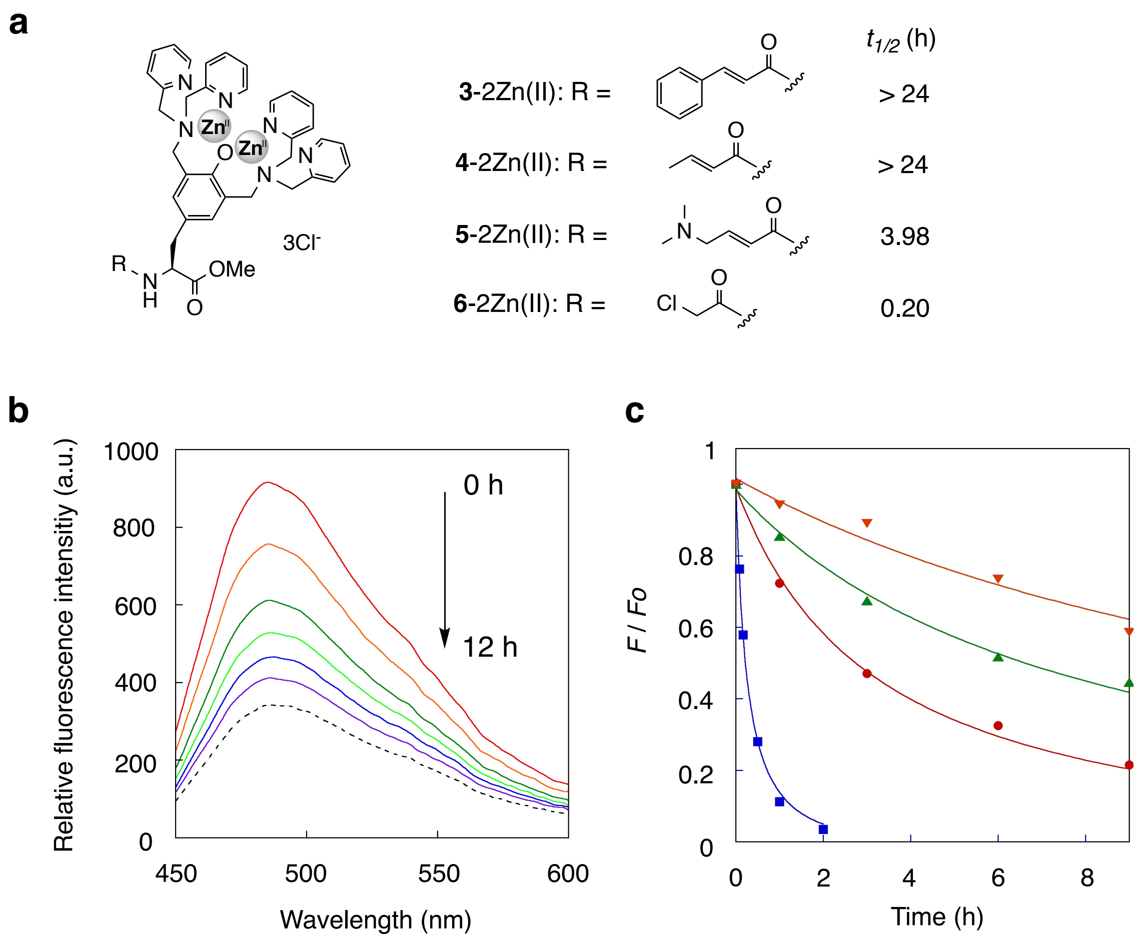

Figure S3. Evaluation of the reactivity of the zinc complexes with peptide-**g**, Related to Table 2. (a) Structures of the zinc complexes and their reaction half-time ($t_{1/2}$, h) with peptide-**g**. (b) Fluorescence intensity change of the solution containing peptide-**g** and **5**-2Zn(II) upon treatment with mCBI (reaction time: 0, 1, 3, 6, 9, and 12 h, from the upper to lower trace). The dashed line indicates the fluorescence of mCBI in the absence of peptide-**g**. Measurement conditions: [peptide-**g**] = 50 μM, [probe] = 100 μM, 50 mM HEPES, 100 mM NaCl, pH 7.2, 37 °C. (c) Plot of fluorescence intensity change for the selected reaction pairs: peptide-**g** and **7**-4Zn(II) (●), peptide-**g** and **8**-4Zn(II) (▼), peptide-**k** and **9**-4Zn(II) (■), and peptide-**i** and **7**-4Zn(II) (▲). Measurement conditions: [peptide] = 50 μM, [probe] = 60 μM, 50 mM HEPES, 100 mM NaCl, pH 7.2, 37 °C.

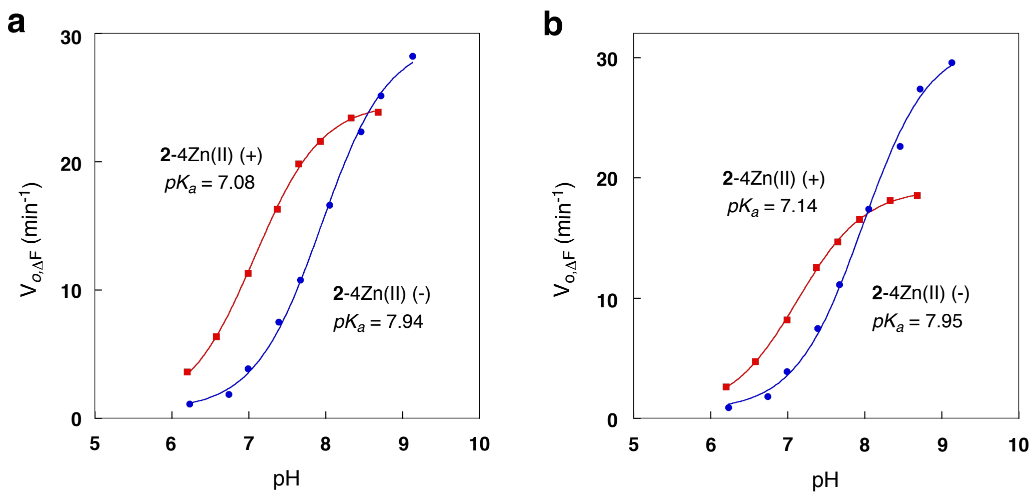

Figure S4. pH-dependent reaction rate profiles of peptide-**j** **(a)** and peptide-**k** (**b**) with mCBI in the presence and absence of **2**-4Zn(II), Related to Table 2. pK_a values were evaluated by applying non-linear curve fitting analysis according to Henderson-Hasselbalch equation.

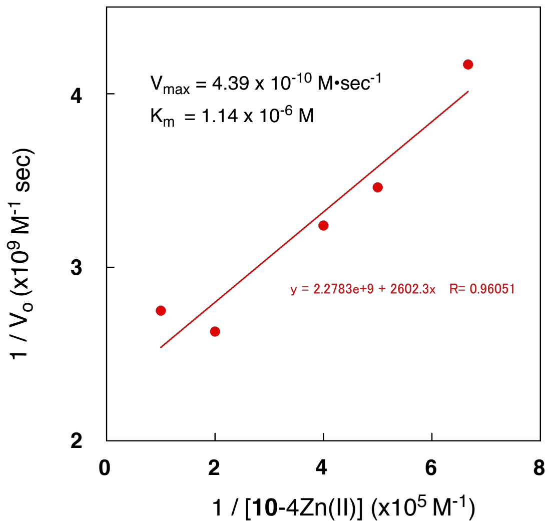

Figure S5. Second-order rate analysis of the labeling reaction of helixD2-tag fused MBP protein with **10**-4Zn(II), Related to Figure 3. The data was analyzed by double reciprocal Lineweaver-Burk plot between the probe concentration (1/[**10**-4Zn(II)]) and initial reaction rate (1/V_o).

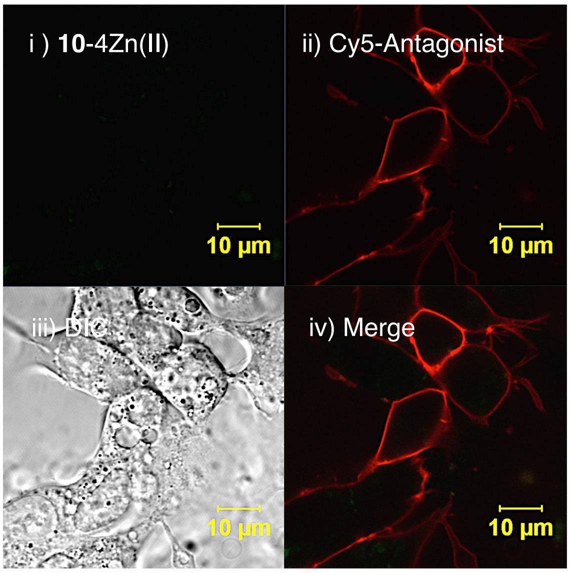

Figure S6. Fluorescence imaging of HEK293 cells expressing helixD2-tag fused B2R upon the labeling with **10**-4Zn(II) in the presence of 1 mM pyrophosphate (PPi), Related to Figure 3. Labeling conditions: [**10**-4Zn(II)] = 4 μM, HEPES-buffered saline, pH 7.4, 37 °C, 30 min.

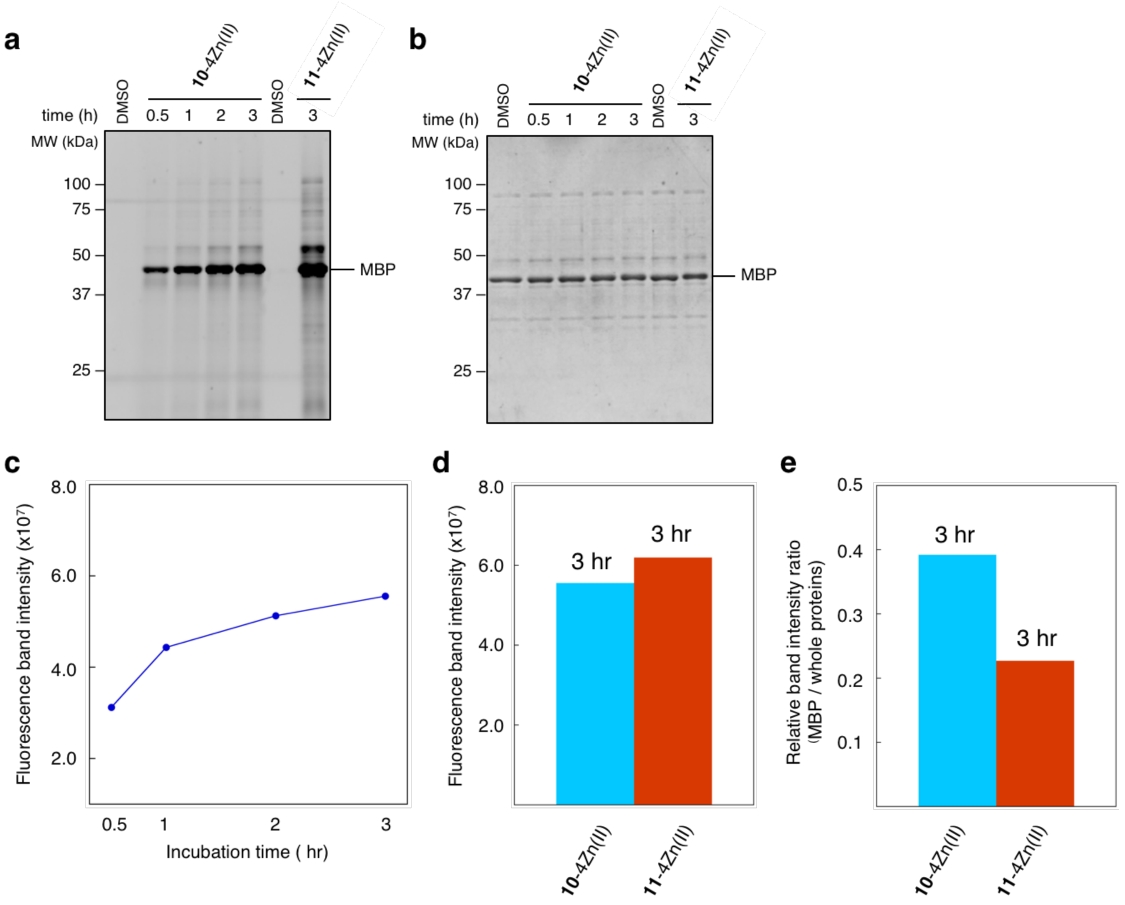

Figure S7. Evaluation of non-specific labeling activity of the probes in E.coli lysate, Related to Figure 3. (a) In-gel fluorescence analysis of helixD2-MBP labeling by **10**-4Zn(II) and **11**-4Zn(I) in the presence of E.coli lysate. (b) CBB image of the gel shown in (a). (c) time-trace plot of the band intensity of helixD2-MBP labeled by **10**-4Zn(II). (d) Comparison of band intensity of helixD2-MBP labeled by **10**-4Zn(II) or **11**-4Zn(II) at 3 hr. (e) Comparison of relative band intensity ratio of helixD2-MBP against whole proteins labeled by **10**-4Zn(II) or **11**-4Zn(II). Note that the relative band intensity ratio of **10**-4Zn(II) is significantly higher than that of **11**-4Zn(II) as shown in (e), while the band intensity of helixD2-MBP labeled by **10**-4Zn(II) was not greatly different from that by **11**-4Zn(II) as shown in (d).

]

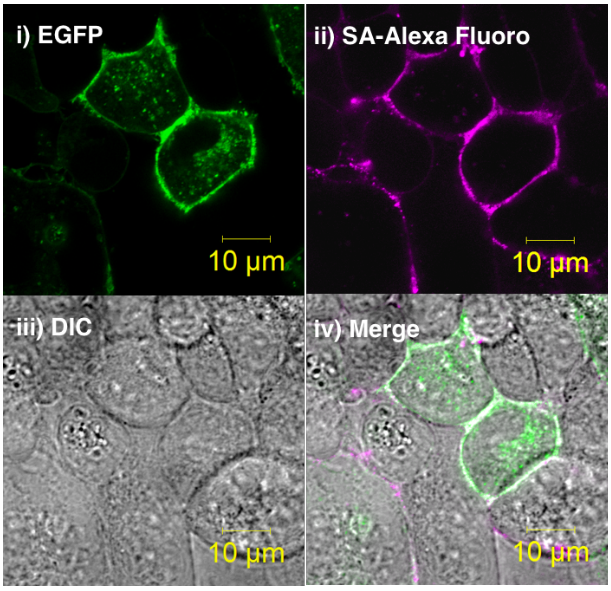

Figure S8. Fluorescence imaging of helixD2-tag fused B2R upon the labeling with **12**-4Zn(II), Related to Figure 4. The labeling reaction was conducted by the same procedure as described for **10**-4Zn(II). After the labeling, the cells were treated with Alexa Fluoro 633-conjugated streptavidin (5 μg/mL in PBS) and subjected to fluorescence imaging.

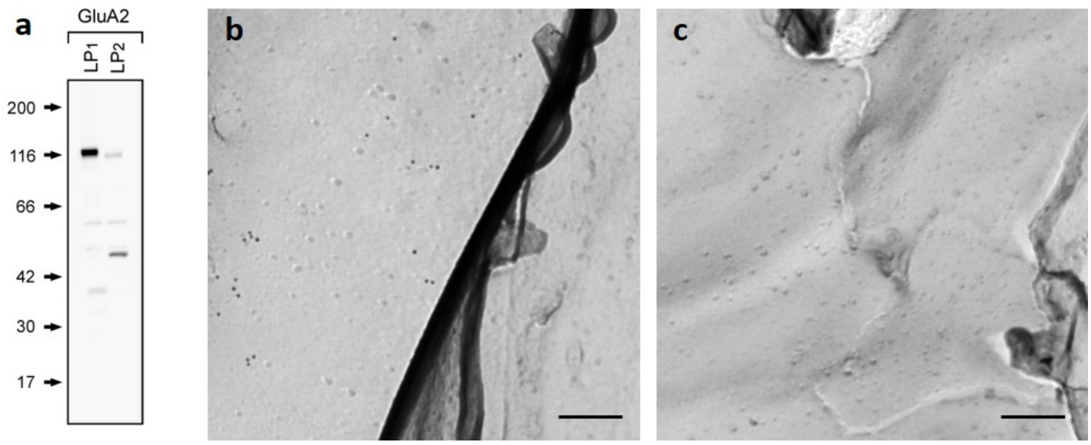

Figure S9. Characterization of rabbit polyclonal antibody for GluA2 AMPA receptor subunit raised against a synthetic peptide (CERDKVNDIVDQVITIGKH), Related to Figure 5. (a) Western-blotting analysis to characterize specificity of the anti-GluA2 antibody. The antibody recognizes a broad band (100-120 kDa) on immunoblots of rat brain LP1 (crude synaptic plasma membrane) and a weak band on LP2 (synaptic vesicle). Subcellular fractionation was performed as described (Yamamori *et al.*, 2011). (b and c) EM images of replicas prepared from GluA2-transfected (b) and non-transfected (c) HEK cells labeled with the anti-GluA2 antibody followed by 10 nm gold-conjugated secondary antibody. Scale bars, 200 nm.

Transparent Methods

Circular Dichroism (CD) Measurement. Aqueous solution of the zinc complex was added to a solution of the peptide (50 μM) in 10 mM borate buffer (pH 8.0). CD spectrum was measured at rt in a quartz cell (0.1 cm path length) using JASCO J-720W spectropolarimeter. The data of each sample was collected by 5 scans (scan range; 350 ~ 190 nm, scan speed; 50 nm•min^{-1}) and averaged to give CD spectrum.

Isothermal Titration Calorimetry (ITC) Measurement. ITC titration was performed with isothermal titration calorimeter (Malvern) at 298 K. Solution of peptide-**c** (6.0 mM) in buffer solution (50 mM HEPES, 100 mM NaCl, pH 7.2) was injected stepwise (5 μL x 48 times) to solution of **1**-2Zn(II) (50 μM) in the same solvent system. The measured heat flow was recorded as function of time and converted into enthalpies (ΔH) by integration of the appropriate reaction peaks. Dilution effects were corrected by subtracting feat flow of blank experiment upon injection of the HEPES buffer under identical experimental conditions. The binding parameters (Kapp, ΔH, ΔS, n) were evaluated by applying one site model using the software Origin (Malvern).

Evaluation of Binding Constant. In a quartz cell, peptide-**b** (0.05 μM, final concentration) dissolved in 3 mL buffer (50 mM HEPES 100 mM NaCl, pH 7.2) was titrated with a freshly prepared aqueous solution of 2-4Zn(II) using a micro syringe at 25 °C. The fluorescence spectrum was measured (λ_{ex} = 410 nm) using Perkin-Elmer LS55 spectrometer at each titration point. The change of the fluorescence intensity at 448 nm was analyzed by nonlinear least-square curve-fitting method to yield the apparent binding constant (K_{app}, M^{-1}).

Kinetic Analysis of Nucleophilic Reaction of Peptide with mCBI. 10 μL of stock solution of mCBI (10 mM in DMSO) was added to a solution of peptide (4 μM) in 0.8 mL of buffer (50 mM HEPES 100 mM NaCl, pH 7.2, degassed under reduced pressure and charged with nitrogen before use) in the presence or absence of 2-4Zn(II) (14 μM). The fluorescence emission at 486 nm was continuously monitored by Perkin-Elmer LS55 spectrometer ((λ_{ex} = 396 nm) at 37 °C. The emission change was analyzed by nonlinear least-square curve-fitting method to yield the first-order reaction constant (k, sec^{-1}). For evaluation of pK_a value of the cysteine residue, initial reaction rate of fluorescence change ($V_{o,\ \Delta F}$, min^{-1}) were measured under the different pH

conditions in universal pH buffer (5 mM CHES-5 mM HEPES-5 mM MES, degassed under reduced pressure and charged with nitrogen before use) at 37 °C. The change of the initial rate over the pH range was analyzed by nonlinear curve-fitting method according to Henderson-Hasselbalch equation to yield pK_a value.

Kinetic Analysis of Cysteine Conjugation of Peptide with Zinc Complex. Peptide (50 μM) and zinc complex (60 μM) was dissolved in 200 □L of buffer solution (50 mM HEPES 100 mM NaCl, pH 7.2, degassed under reduced pressure and charged with nitrogen before use) and incubated at 37 °C in a plastic tube. Aliquot of the solution (20 μL) was sampled at appropriate times and mixed with 6 μL of inorganic pyrophosphate solution (10 mM in water) to quench the reaction followed by being kept at -20 °C. The thawed samples were mixed with 4 μL of mCBI (5 mM in DMSO) and incubated at 37 °C for 1 h in a dark. The solution (18μL) was diluted with a 582 μL of buffer solution (50 mM HEPES 100 mM NaCl, pH 7.2), and the fluorescence was measured (λ_{ex} = 396 nm) in a quartz cell using Perkin-Elmer LS55 spectrometer. The fluorescence change (λ_{em} = 486 nm) was analyzed by nonlinear least-square curve-fitting method according to second-order equation to yield half-reaction time ($t_{1/2}$, h).

Fluorescence Detection of Covalent Labeling of Tag-fused MBP. A solution of helixD2-tag-fused MBP (1 μM) or CH6 tag-fused MBP (1 μM) in 50 mM HEPES, 100 mM NaCl, 20 μM TCEP, pH 7.2 (degassed under reduced pressure and charged with nitrogen before use) was mixed with **10**-4Zn(II) (10 μM, final concentration), and the mixture was incubated in a plastic tube at 37 °C. Aliquot of the solution (8 μL) was sampled at the appropriate times and mixed with 2 μL inorganic pyrophosphate solution (10 mM in water) followed by being kept at -20 °C. After thawing, the samples were mixed with 5x sampling buffer and heated at 95 °C for 2 min followed by being applied to SDS-PAGE. In-gel fluorescence and CBB analyses were performed with LAS-4000 lumino image analyzer (FUJIFILM) by EPI (460 nm excitation, Y515Di filter) and DIA-W mode, respectively.

Cell Culture and B2R Expression in HEK293 Cells. HEK293 cells were cultured in high glucose DMEM supplemented with 10% fetal bovine serum (FBS), penicillin (100 units/mL), streptomycin (100 μg/mL) and amphotericin B (250 ng/mL). Cells were maintained at 37 ºC in humidified atmosphere of 5% CO_2 in air. Subculture was performed every 3-4 days from subconfluent (< 80%) cultures using trypsin-EDTA solution. Transfection of the expression

vector for B2R was carried out in a 35 mm glass-bottomed dish (Iwaki) using Lipofectamine LTX (Invitrogen) according to the general procedure. The cells were subjected to labeling experiment after 48 h of the transfection.

Fluorescence Imaging of Tag-fused B2R. In a glass bottom dish, HEK293 cells (~ 1 x 10^6) transiently expressing helixD2-tag fused B2R or CH6 tag-fused B2R were incubated in non-serum Dulbecco's modified Eagle medium (DMEM) containing 2-deoxy-D-glucose (10mM) and sodium azide (6 mM) for 30 min at 37 °C in CO_2 incubator. After removal of DMEM, cells were treated with TCEP (1 mM) for 10 min at rt in 1 mL HEPES-buffered saline (HBS, containing 107 mM NaCl, 6 mM KCl, 1.2 mM $MgSO_4$, 2 mM $CaCl_2$, 11.5 mM glucose, 10 mM 2-deoxy-D-glucose, 6 mM sodium azide 20 mM HEPES, adjusted to pH 7.4 with NaOH). TCEP solution was removed and then cells were treated with **10**-4Zn(II) (4 μM, final concentration) for 30 min in HBS containing 2-deoxy-D-glucose (10 mM) and sodium azide (6 mM) at 37 °C. Cells were washed three times with HBS containing 2 mM pyrophosphate (PPi) (1 mL) to remove unreacted **10**-4Zn(II). HBS containing Cy5-appended B2R antagonist peptide (0.4 μM) was added and cells were analyzed by confocal laser scanning microscope (Zeiss, LSM700) using appropriate laser and emission filter settings for Oregon Green 488 (λ_{ex} =488 nm, λ_{em} = 490-555 nm) and Cy5 (λ_{ex} = 639 nm, λ_{em} > 640 nm).

Evaluation of Non-specific Labeling Property of Probe. In a glass bottom dish, HEK293 cells (~ 1 x 10^6) were incubated in non-serum DMEM containing 2-deoxy-D-glucose (10mM) and sodium azide (6 mM) for 30 min at 37 °C in a CO_2 incubator. After removal of DMEM, cells were treated with TCEP (1 mM) for 10 min at rt in 1 mL HEPES-buffered saline. TCEP solution was removed and then cells were treated with **10**-4Zn(II) or **11**-4Zn(II) (4 μM, final concentration) for 10 or 30 min in HBS containing 2-deoxy-D-glucose (10 mM) and sodium azide (6 mM) at 37 °C. After washing three times with HBS containing 2 mM PPi (1 mL), the cells were fixed by treatment with PBS (100 μL) containing 4% paraformaldehyde, 0.2% glutaraldehyde, and 2 mM PPi for 10 min with gentle shaking (80 rpm) at rt. The fixed cells were washed three times with HBS containing 0.1% Triton-X and 2 mM pyrophosphate (PPi) (1 mL) by gentle shaking (80 rpm) for 10 min at rt. The cells were immersed in HBS (100 μL) and the fluorescence on the cell surfaces were detected by confocal laser scanning microscope (Leica, TCS SP8) equipped with HyD detector with a high laser power (1%) for Oregon Green 488 (λ_{ex}

=488 nm). The fluorescence image was analyzed by ImageJ software (National Institutes of Health) to obtain the fluorescence intensity per area on the cell surface.

EM Detection of B2R in Freeze-fracture Replicas. HEK293T cells (ca. 2×10^7 cells) transiently expressing helixD2-B2R-EGFP, GluA2 (for negative control) or co-expressing helixD2-B2R-EGFP and GluA2 were washed with HEPES-buffered saline (HBS), and treated with 2 mM TCEP in HBS (1 mL) for 10 min at 37 °C. After removal of TCEP solution, the cells were treated with 3 μM **12**-4Zn(II) in HBS for 30 min at 37 °C, or with 3 μM **13**-4Zn(II) in HBS for 2 h at 37 °C. After washing with HBS containing 2 mM PPi, the cells labeled with **12**-4Zn(II) were incubated with 1.4-nm gold particle-conjugated streptavidin (Nanoprobes) for 1 h at 37 °C. For estimation of chemical labeling density in unfixed cells, cells were fixed after chemical labeling (after washing out the 13-4Zn(II) with PPi) and for estimation of chemical labeling density in fixed cells, fixation was performed before chemical labeling (before HBS washing). Fixation was done with 2% paraformaldehyde in phosphate-buffered saline (PBS) for 15 min at RT. The cells were harvested by scraping, collected by centrifugation (100g, 5 min), and the cell pellet was sandwiched between gold carriers for high-pressure freezing (HPM010, Bal-Tec). The frozen pellet was then fractured into two parts at –120 °C and replicated by depositions of 30 nm carbon layer using freeze-fracture replica machine (BAF 060, Bal-Tec). After thawing, the replicas were washed with 2.5% SDS, 20% sucrose, and 15 mM Tris-HCl (pH 8.3) under the following conditions: 48 h at 60 °C for labeling with **13**-4Zn(II); 3 d at 60 °C for labeling of pre-fixed cells with **12**-4Zn(II); and 18 h at 60 °C for labeling with **12**-4Zn(II).

For EM detection of 1.4-nm gold particle-conjugated streptavidin, the replicas were treated with reagents from a silver intensification kit (HQ Silver, Nanoprobes). For immunolabeling, the replicas were treated with mouse anti-GFP (CLONTECH), rabbit anti-FLAG (Sigma-Aldrich) or rabbit anti-GluA2 antibodies (Figure S9) for 48 h at 15 °C, followed by staining with anti-mouse secondary antibody conjugated with 5-nm gold particles or anti-rabbit secondary antibody conjugated with 10-nm gold particles (British Biocell International). The silver-intensified replicas were analyzed by EM using Tecnai 10/12 transmission electron microscope (FEI); those without silver intensification were analyzed by JEM-2800 scanning transmission electron microscope (JEOL) in dark field mode. For quantitative analysis of particle distribution, we used GPDQ software (Wickman *et al.*, 2018) to calculate the density and NND of particles, particle number per cluster, and cluster area. For the definition of clusters of particles, we used 3 particles as a minimum number and mean + 2SD (Miki *et al.*, 2017; Wickman *et al.*, 2018) of

fitted peaks of NND (Figure 5I) as a maximum distance allowed (6 + 3×2 = 12 nm for chemical labeling, 22 + 10×2 = 42 nm for immunolabeling).

EM Detection of B2R in Embedded Ultrathin Sections. HEK293 cells (*ca.* 2×10^7 cells) transiently expressing helixD2-B2R-EGFP cultured on a coverslip were treated with 2 mM TCEP in HBS for 10 min at 37 °C. After removal of the solution, the cells were treated with 3 μM **13-**4Zn(II) for 2 h at 37 °C, and washed with 2 mM PPi in HBS. The cells were then fixed in PBS containing 4% paraformaldehyde and 0.05% glutaraldehyde for 10 min at rt. After 3-6 min silver intensification, the cells were treated with 0.2% OsO_4 in PBS for 20 min, counterstained with 0.25% uranyl acetate overnight at 4 °C, dehydrated in ascending series of EtOH and propylene oxide, and embedded in Durcupan (Sigma-Aldrich). Ultrathin sections (70-μm thick) prepared using ultramicrotome (Leica Ultracut UCT, Leica) were counterstained with lead citrate and uranyl acetate, and analyzed by Tecnai 10 transmission electron microscope (FEI). For immunolabeling of the FLAG tag, HEK293 cells were fixed by 4% paraformaldehyde and 0.05% glutaraldehyde in PBS for 10 min at rt. After blocking with 10% normal goat serum and 2% bovine serum albumin in Tris-buffered saline (TBS), the cells were treated with anti-FLAG antibody (Sigma-Aldrich) followed by anti-mouse secondary antibody conjugated with 1.4-nm gold particles (Nanoprobes). After 3-6 min silver intensification, the cells were treated, sectioned and observed in the same way as described above. To measure the distance between gold particles and cell membrane, we first tilted the ultrathin sections to obtain perpendicular views of lipid bilayer (Figure 6B and 6D). We discarded the particles if it was not possible to obtain perpendicular views. Then, the distance between the center of silver-intensified gold particles and the midpoint of plasma membrane was determined by ImageJ v1.51 software (National Institutes of Health).

Construction of expression-vectors of the tag-fused maltose binding protein (MBP)

DNA encoding helixD2-tag (KKCPYSAADAAADAAADAAAD) was introduced into the pMAL-c2X vector (New England BioLabs) encoding D4-tag fused MBP (Nonaka *et al.*, 2010) by inverse polymerase chain reaction (PCR). After digestion of the template plasmid by the treatment with *Dpn* I, 5'-termini of the PCR product were phosphorylated by T4 polynucleotide kinase and then cyclized by DNA ligase to give the vector encoding helixD2-tag fused MBP. The DNA sequence of the vectors was confirmed by 3130xl Genetic Analyzer (Applied Biosystems).

Expression and purification of tag-fused MBP

pMAL-c2X vector encoding helixD2-tag fused MBP was transformed into T7 Expression *lysY/I^q^* competent *E. coli* (New England BioLabs). Single colony was cultured at 37 °C in 2YT media (200 mL) containing ampicillin (100 mg/L) at 37 °C until optical density (OD) at 600 nm increased to 0.6. and further grown at 37 °C for 6 h with IPTG induction (1 mM final conc.). Cells were spun down for 10 min at 13,500 rpm and re-suspended in equilibrium buffer (20 mM Tris–HCl, 200 mM NaCl, 1mM EDTA, 1mM DTT, pH 8.0) and then lysed by sonication (150 shots x 2) on ice. Insoluble materials were removed by centrifugation (13,500 rpm, 10 min) and supernatant was applied to a MBPTrap affinity column (GE Healthcare). Column was washed with equilibrium buffer (x2) and then MBP was eluted by elusion buffer (20 mM Tris–HCl, 200 mM NaCl, 10 mM maltose, pH 8.0). Purity of MBP in fractions was checked by SDS-PAGE. Concentration of purified MBP was determined by the UV absorbance at 280 nm using the calculated extinction coefficient (ε_{280nm} = 69,330 $M^{-1}cm^{-1}$). MBP solution was kept at 4 °C in the presence of 1 mM TCEP.

Construction of vectors of tag-fused B2R receptor

DNA encoding helixD2-tag (KKCPYSAADAAADAAADAAAD) or CH6 tag inserted into pCIneo plasmid encoding α7-D4-B2R vector (Nonaka *et al.*, 2010) by inverse polymerase chain reaction (PCR). After digestion of template plasmid by the treatment with *Dpn* I, 5'-termini of the PCR product were phosphorylated by T4 polynucleotide kinase and then cyclized by DNA ligase to give the vector encoding α7-helixD4-B2R or α7-CH6-B2R. DNA sequences were confirmed by 3130xl Genetic Analyzer (Applied Biosystems).

Solid-Phase Peptide Synthesis

Peptides except for peptide-**c** were synthesized by solid-phase peptide synthesis using standard Fmoc-based coupling chemistry. Coupling reactions (0.2 mmol scale) were performed using 4 equiv of amino acid, 4 equiv of HOBt and 4 equiv of HBTU on Rink Amide MBHA Resin LL (0.2 mmol, 0.38 mmol/g, Merck Millipore). *N*-terminal was acetylated by treatment with 20 equiv of Ac_2O in NMP. Peptide cleavage and deprotection were carried out by treatment of resin with TFA / H_2O / triisopropylsilane (95 : 2.5 : 2.5) for 3 h at rt. The crude peptide was collected by precipitation in *tert*-butyl methyl ether and purified by reverse-phase HPLC (column; YMC-pack ODS-A, 20 x 250 mm) using CH_3CN (0.1% TFA) / H_2O (0.1% TFA) solvent system with a linear gradient mode. Typical HPLC conditions are as follows; 5 / 95 (0 min) →5 / 95 (10 min) → 40 / 60 (50 min), flow rate 9.9 mL/min, UV detection at 220 nm. Purified peptide was lyophilized and stocked in a refrigerator (-30 °C).

MALDI-TOF-MS analysis (CHCA)

peptide-**a**: calcd for $C_{57}H_{84}N_{16}O_{25}$ (MW; 1392.6), found 1416.3 $[M+Na]^+$.

peptide-**b1**[a]: calcd for $C_{54}H_{87}N_{17}O_{24}$ (MW; 1357.6), found 1358.7 $[M+H]^+$.

peptide-**d**: calcd for $C_{68}H_{102}N_{20}O_{29}S$ (MW; 1694.7), found 1694.3 $[M]^+$.

peptide-**e**: calcd for $C_{69}H_{102}N_{20}O_{29}S$ (MW; 1706.7), found 1706.8 $[M]^+$.

peptide-**f**: calcd for $C_{70}H_{104}N_{20}O_{29}S$ (MW; 1720.7), found 1719.8 $[M-H]^-$.

peptide-**g**: calcd for $C_{77}H_{120}N_{22}O_{30}S$ (MW; 1864.8), found 1864.2 $[M-H]^-$.

peptide-**h**: calcd for $C_{77}H_{120}N_{26}O_{30}S$ (MW; 1920.8), found 1920.2 $[M-H]^-$.

peptide-**i**: calcd for $C_{68}H_{95}N_{19}O_{36}S$ (MW; 1785.6), found 1783.1 $[M-H]^-$.

peptide-**j**: calcd for $C_{80}H_{125}N_{23}O_{31}S$ (MW; 1935.9), found 1937.0 $[M]^-$.

peptide-**k**: calcd for $C_{83}H_{130}N_{24}O_{32}S$ (MW; 2006.9), found 2005.5. $[M-H]^-$.

[a)] peptide-**b1** is a peptide that lacks 7-hydroxycumarin from peptide-**b**.

Conjugation of peptide-b1 with 7-hydroxycumarine

A solution of the peptide-**b1** (3 mg, 2.21 μmol), 7-hydroxycumarine succinimidyl ester (3.3 mg, 11.1 μmol) and DIEA (438 μL, 1.16 mmol) in dry DMF (2 mL) was stirred overnight at rt. H_2O (1.5 mL) was added, the solution was stirred for 1 h at rt. Purification was conducted by HPLC (column; YMC-Actus Triart C18, 20 x 250 mm) using CH_3CN (0.1% TFA) / H_2O (0.1%

TFA) solvent system with a linear gradient mode. The collected fractions were lyophilized to give peptide-**b**.

HPLC conditions: column; YMC-Actus Triart C18, 250 x 20 mm, mobile phase; CH_3CN (containing 0.1% TFA) / H_2O (containing 0.1% TFA) = 5 / 95(0-10 min) → 60 / 40 (linear gradient over 50 min), MALDI-TOF-MS: calcd for $C_{64}H_{91}N_{17}O_{28}$ 1545.6, found 1544.5 $[M-H]^-$.

General Materials and Methods for Organic Synthesis

Unless otherwise noted, chemical reagents were purchased from commercial suppliers (Tokyo Chemical Industry (TCI), Wako Pure Chemical Industries, Watanabe Chemical Industries, and Sigma-Aldrich) and used without further purification. ^{1}H-NMR spectra were recorded using Varian UNITY-400 (400 MHz) spectrometer, and chemical shifts (δ, ppm) were referenced to residual solvent peak. ESI and MALDI-TOF mass spectra were measured using Bruker micrOTOF II and Bruker autoflex III spectrometer, respectively. HPLC purification was conducted with Hitachi L-7100. Compound **6**, **15** and **18** were synthesized according to the previous report (Nonaka *et al.*, 2010).

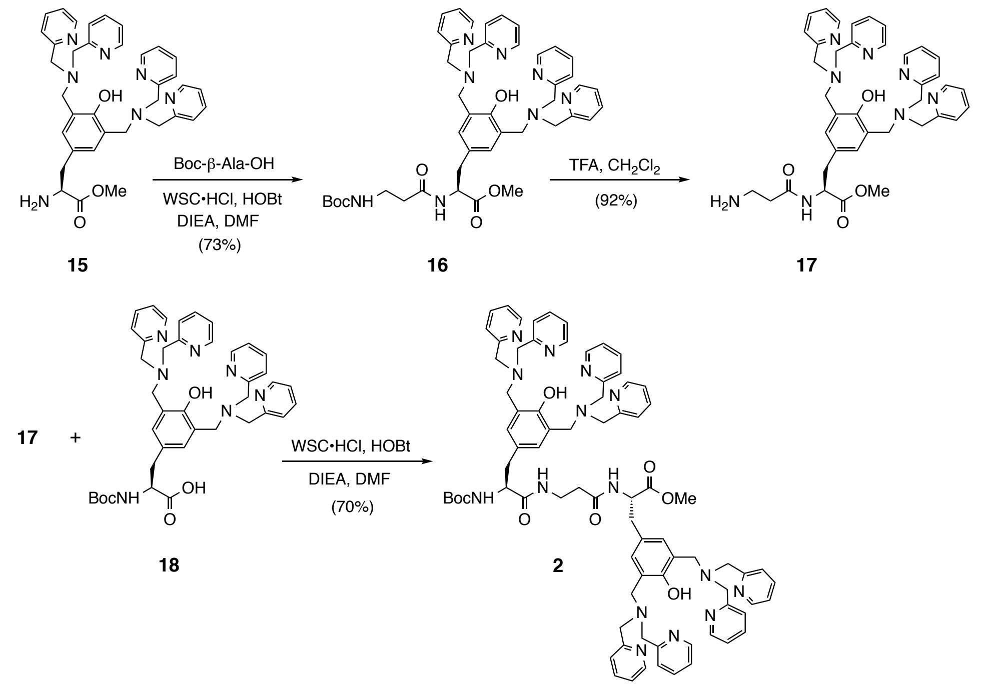

Synthesis of **16**

To a solution of Boc-βAla-OH (185 mg, 0.98 mmol) in dry DMF (5 mL) was added WSCI·HCl (188 mg, 0.98 mmol), HOBt·H_2O (150 mg, 0.98 mmol), DIEA (391 μL, 2.4 mmol) and **15** (433 mg, 0.70 mmol). The solution was stirred overnight at rt. After dilution with AcOEt, the mixture was washed with saturated $NaHCO_3$ and brine followed by drying over Na_2SO_4. After removal of the solvent by evaporation, the residue was purified by column chromatography on SiO_2 ($CHCl_3$: MeOH : NH_3 = 400 : 10 : 1 → 300 : 10 : 1) to give **16** (410 mg, 73%) as a colorless amorphous powder.

^{1}H-NMR(400 MHz, $CDCl_3$): δ 10.99 (1H, br), 8.50 (4H, s), 7.56 (4H, t, *J* = 7.4 Hz), 7.42 (4H, d, *J* = 7.6 Hz), 7.09 (4H, t, *J* = 5.8 Hz), 6.94 (2H, s), 6.71 (1H, d, *J* = 8.0 Hz), 5.26 (1H, br), 4.74

(1H, m), 3.83 (8H, s), 3.75 (4H, s), 3.65 (3H, s), 3.25 (2H, d, *J* = 5.6 Hz), 3.01 (2H, t, *J* = 8.8 Hz), 2.21 (2H, m), 1.37 (9H, s). ESI-TOF-MS *m/z* $[M + Na]^+$ calcd for $C_{44}H_{52}N_8O_6Na$ 811.39; Found 811.39.

Synthesis of **17**

To an ice-cooled solution of **16** (410 mg, 510 μmol) in CH_2Cl_2 (2.5 mL) was added dropwise TFA (2.5 mL) and the solution was stirred for 1 h at rt. The solvent was removed in vacuo and the residue was dissolved in H_2O. The mixture was neutralized with aq. NH_3 on ice-cooling and then extracted with CH_2Cl_2. The organic layer was washed with brine followed by drying over Na_2SO_4. The solution was concentrated by evaporation to give **17** (330 mg, 92%) as a colorless amorphous powder.

^{1}H-NMR (400 MHz, $CDCl_3$) : δ 10.90 (1H, br), 8.47 (4H, s), 8.25 (1H, br), 7.51 (4H, t, *J* = 7.4 Hz), 7.30 (4H, d, *J* = 7.6 Hz), 7.07 (4H, t, *J* = 6.0 Hz), 6.93 (2H, s), 4.66 (1H, br), 3.86 (4H, m), 3.65 (11H, m), 2.93 (4H, m), 2.59 (1H, br), 2.33 (1H, m). ESI-TOF-MS *m/z* $[M + Na]^+$ calcd for $C_{39}H_{45}N_8O_4Na$ 689.36; Found 689.36.

Synthesis of **2**

To a solution of **18** (430 mg, 0.61 mmol) in dry DMF (5 mL) was added WSCI•HCl (115 mg, 0.61 mmol), HOBt•H_2O (93 mg, 0.61 mmol), DIEA (246 μL, 1.41 mmol) and **17** (330 mg, 0.47 mmol). The solution was stirred overnight at rt. After dilution with AcOEt, the mixture was washed with saturated $NaHCO_3$ and brine followed by drying over Na_2SO_4. After removal of the solvent by evaporation, the residue was purified by column chromatography on SiO_2 ($CHCl_3$: MeOH : NH_3 = 300 : 10 : 1→200 : 10 : 1) to give **2** (450 mg, 70%) as a colorless amorphous powder.

^{1}H-NMR(400 MHz, $CDCl_3$): δ 10.95 (2H, br), 8.47 (8H, s), 7.56 (8H, m), 7.40 (8H, t, *J* = 6.4 Hz), 7.14 (1H, br), 7.04 (10H, m), 6.95 (2H, s), 5.36 (1H, br), 4.65 (1H, m), 4.23 (1H, br), 3.73 (25H, m), 3.57 (3H, s), 3.46 (1H, s), 3.16 (1H, br), 2.91 (4H, m), 2.11 (2H, m), 1.24 (9H, s). ESI-TOF-MS 1396.66 $[M + H]^+$. ^{13}C NMR (125 MHz, $CDCl_3$) δ 173.1, 172.1, 172.0, 159.1, 155.7, 155.1, 155.0, 149.0, 136.9, 130.4, 130.3, 126.5, 126.0, 124.0, 123.3, 123.3, 122.2 x 2, 79.7, 59.7, 56.4, 54.9, 54.8, 54.1, 52.3, 37.9, 36.5, 35.9, 35.8, 28.3. ESI-TOF-MS *m/z* $[M + Na]^+$ calcd for $C_{79}H_{87}N_{15}O_8Na$ 1396.68; Found 1396.66.

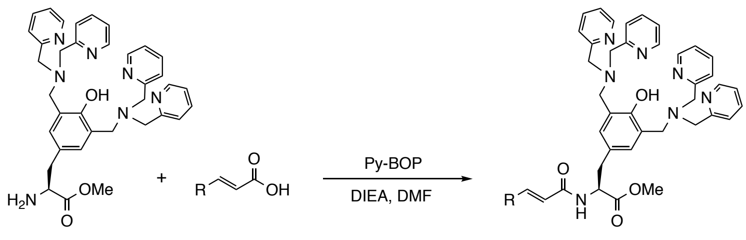

15

3: R = Ph (86%)
4: R = CH_3 (21%)
5: R = $CH_2N((CH_3)_2$ (95%)

Synthesis of **3**

To an ice-cooled solution of cinnamic acid (12 mg, 81 μmol) in dry DMF (1.5 mL) was added triethylamine (11 μL, 81 μmol), Py-BOP (36 mg, 81 μmol) and **15** (50 mg, 81 μmol). The solution was stirred for 3 h at rt. After dilution with AcOEt, the mixture was washed with saturated $NaHCO_3$ and brine followed by drying over Na_2SO_4. After removal of the solvent by evaporation, the residue was purified by column chromatography on SiO_2 ($CHCl_3$: MeOH : NH_3 = 400 : 10 : 1) to give **3** (52 mg, 86%) as a colorless amorphous powder.

^{1}H-NMR(400 MHz, DMSO-d_6): δ 10.94 (1H, s), 8.49-8.45 (5H, m), 7.68 (4H, t, *J* = 7.2 Hz), 7.44-7.32 (9H, m), 7.20 (4H, t, *J* = 6.0 Hz), 7.05 (2H, s), 6.65 (1H, d, *J* = 16 Hz), 4.6 (1H, q, *J* = 13.6 Hz, 6.4 Hz), 3.73 (8H, s), 3.64 (4H, s), 3.52 (3H, s), 2.98-2.85 (1H, m). ESI-TOF-MS *m/z* [M + H]$^+$ calcd for $C_{45}H_{46}N_7O_4$ 748.36; Found 748.38.

Synthesis of **4**

To an ice-cooled solution of crotonic acid (4.1 mg, 48 μmol) in dry DMF (1.5 mL) was added a mixture of triethylamine (6.7 μL, 48 μmol), Py-BOP (22 mg, 48 μmol) and **15** (30 mg, 48 μmol). The solution was stirred for 28 h at rt. After dilution with AcOEt, the mixture was washed with saturated $NaHCO_3$ and brine followed by drying over Na_2SO_4. After removal of the solvent by evaporation, the residue was purified by column chromatography on SiO_2 ($CHCl_3$: MeOH : NH_3 = 250 : 10 : 1) to give a pale yellow oil (24 mg). This oil was further purified by column chromatography on SiO_2 ($CHCl_3$: MeOH : NH_3 = 350 : 10 : 1) to give **4** (7 mg, 21%) as a pale yellow oil.

^{1}H-NMR(400 MHz, $CDCl_3$): δ 10.94 (1H, br), 8.52 (4H, d, *J* = 4.8 Hz), 7.60 (4H, t, *J* = 7.6 Hz), 7.44 (4H, d, *J* = 8.0 Hz), 7.11 (4H, t, *J* = 6.4 Hz), 6.97 (2H, s), 6.80-6.74 (1H, m), 6.41 (1H, d, *J* = 7.6 Hz), 4.86 (1H, q, *J* = 13.6 Hz, 7.6 Hz), 3.85 (8H, s), 3.76 (4H, s), 3.65 (3H, s), 3.05 (2H, d, *J* = 5.2 Hz), 1.68 (3H, d, *J* = 6.8 Hz). ESI-TOF-MS *m/z* [M + H]$^+$ calcd for $C_{40}H_{44}N_7O_4$ 686.34; Found 686.36.

Synthesis of **5**

To an ice cooled solution of 4-(dimethylamino)-2-butenoic acid hydrochloride (13 mg, 80 μmol) in dry DMF (1.5 mL) was added DIEA (44 μL, 240 μmol), Py-BOP (36 mg, 80 μmol) and **15** (50 mg, 80 μmol). The solution was stirred for 44 h at rt. After dilution with AcOEt, the mixture was washed with saturated $NaHCO_3$ and brine followed by drying over Na_2SO_4. After removal of the solvent by evaporation, the residue was purified by column chromatography on SiO_2 ($CHCl_3$: MeOH : NH_3 = 300 : 10 : 1→150 : 10 : 1) to give **5** (56 mg, 95%) as a pale yellow oil.

^{1}H-NMR(400 MHz, $CDCl_3$): δ 8.52 (4H, d, J = 4.8 Hz), 7.58 (4H, t, J = 8.0 Hz), 7.44 (4H, d, J = 8.0 Hz), 7.10 (4H, t, J = 6.0 Hz), 6.97 (2H, s), 6.80-6.74 (1H, m), 6.57 (1H, d, J = 7.6 Hz), 4.86 (1H, q, J = 13.2 Hz, 5.6 Hz), 3.84 (8H, s), 3.76 (4H, s), 3.65 (3H, s), 3.05 (2H, d, J = 6.0 Hz), 2.89 (2H, m), 2.14 (6H, s). ESI-TOF-MS *m/z* $[M + H]^+$ calcd for $C_{42}H_{49}N_8O_4$ 729.39; Found 729.40.

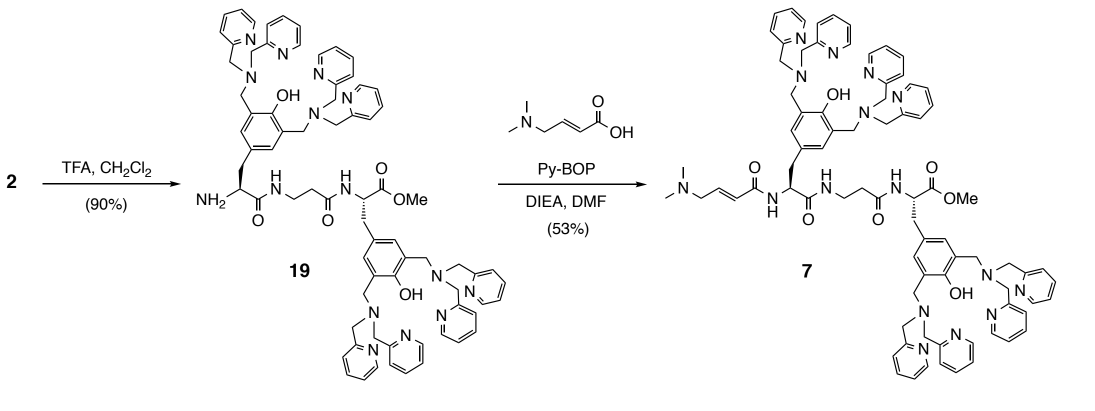

Synthesis of **19**

To an ice-cooled solution of **2** (300 mg, 0.24 mmol) in dry CH_2Cl_2 (2.0 mL) was added dropwise TFA (2.5 mL) and the solution was stirred for 1 h at rt. After removal of the solvent in vacuo, the residue was dissolved in H_2O. The mixture was neutralized with aqueous NH_3 on ice-cooling and then extracted with CH_2Cl_2. The organic layer was washed with brine followed by drying over Na_2SO_4. The solution was concentrated by evaporation to give **19** (250 mg, 90%) as a colorless amorphous powder.

^{1}H-NMR (400 MHz, $CDCl_3$) : δ 10.96 (2H, br), 8.50 (8H, t, J = 2.4 Hz), 7.76 (1H, m), 7.59 (8H, t, J = 7.6 Hz), 7.45 (8H, t, J = 8.0 Hz), 7.10 (8H, t, J = 6.0 Hz), 7.03 (2H, s), 6.97 (2H, s), 4.77 (1H, dd, J = 6.8 Hz), 3.84 (16H, s), 3.80-3.73 (9H, m), 3.62 (3H, s), 3.44-3.35 (4H, m), 3.11-2.98

(4H, m), 2.56-2.50 (1H, m), 2.25-2.24 (2H, m). ESI-TOF-MS *m/z* $[M + H]^+$ calcd for $C_{74}H_{80}N_{15}O_6$ 1274.64; Found 1274.66.

Synthesis of **7**

To an ice-cooled solution of 4-(dimethylamino)-2-butenoic acid hydrochloride (3.8 mg, 25 μmol) in dry DMF (1.0 mL) was added DIEA (12 μL, 63 μmol), Py-BOP (12 mg, 24 μmol) and **19** (20 mg, 16 μmol). The solution was stirred for 38 h at rt. After dilution with AcOEt, the solution was washed with saturated $NaHCO_3$ and brine followed by drying over Na_2SO_4. After removal of the solvent by evaporation, the residue was purified by column chromatography on SiO_2 ($CHCl_3$: MeOH : NH_3 = 400 : 4 : 1→180 : 30 : 1) to give **7** (12 mg, 53%) as a colorless oil. ^{1}H-NMR (400 MHz, DMSO-d_6) : δ 10.93 (1H, s), 10.81 (1H, s), 8.45 (8H, d, *J* = 4.8 Hz), 8.30 (1H, d, *J* = 9.2 Hz), 8.12 (1H, d, *J* = 8.4 Hz), 7.98 (1H, m), 7.68 (8H, t, *J* = 7.2 Hz), 7.42 (8H, d, *J* = 7.6 Hz), 7.20 (8H, dd, *J* = 1.4 Hz, 6.2 Hz), 7.03 (2H, s), 6.99 (2H, s), 6.36-6.33 (1H, m), 5.96 (1H, d, *J* = 15.6 Hz), 4.50-4.40 (2H, m), 3.71 (16H, d, *J* = 8.4 Hz), 3.61 (8H, d, *J* = 11.2 Hz), 3.42 (3H, s), 3.15-3.14 (4H, m), 2.88-2.62 (8H, m), 2.24-2.14 (4H, m), 1.93 (6H, s). ESI-TOF-MS *m/z* $[M + H]^+$ calcd for $C_{80}H_{19}N_{16}O_7$ 1385.71; Found 1385.72.

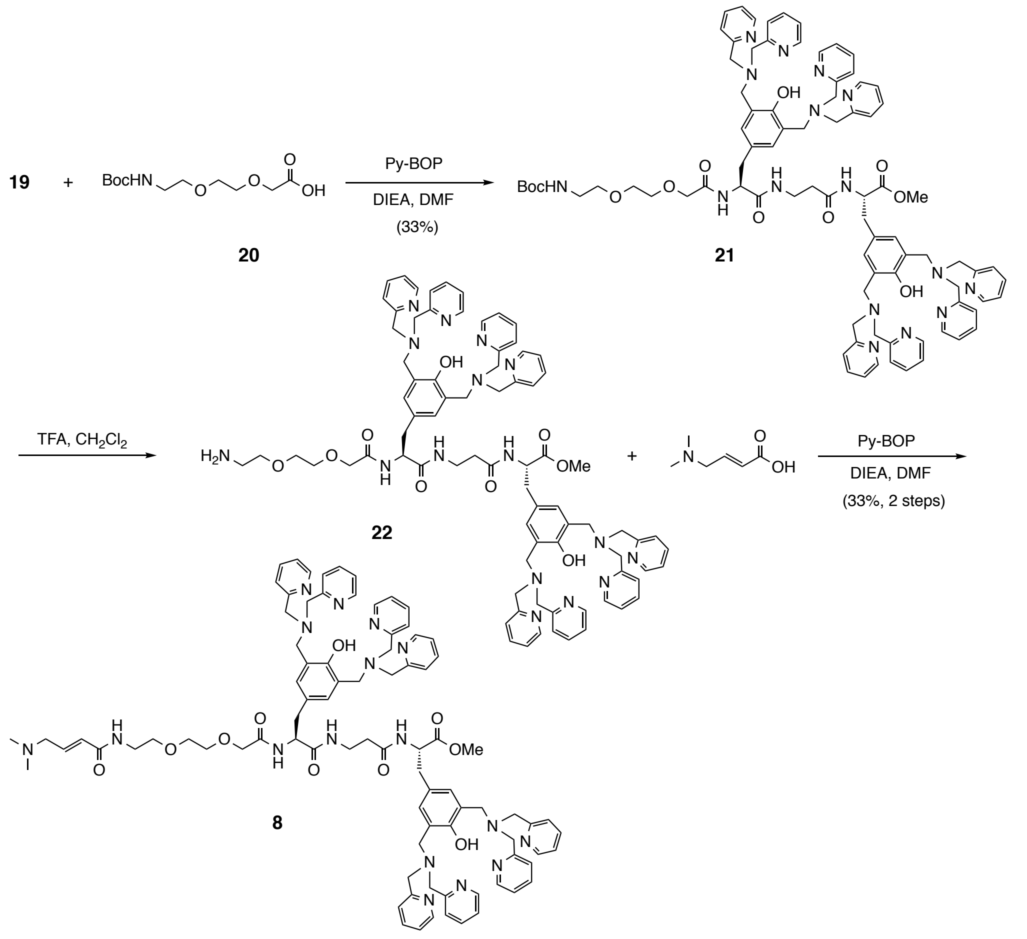

Synthesis of **21**

To an ice-cooled solution of **20** (13 mg, 47 μmol) in dry DMF (1.5 mL) was added DIEA (23 μL, 126 μmol), Py-BOP (25 mg, 47 μmol) and **19** (40 mg, 31 μmol) and the solution was stirred for 13 h at rt. After dilution with AcOEt, the solution was washed with saturated $NaHCO_3$ and brine followed by drying over Na_2SO_4. After removal of the solvent by evaporation, the residue was purified by column chromatography on SiO_2 ($CHCl_3$: MeOH : NH_3 = 400 : 4 : 1→160 : 20 : 1) to give **21** (44 mg, 33%) as a colorless oil. This material contained a small amount of impurities but was used for the next reaction without further purification.

^{1}H-NMR (400 MHz, DMSO-d$_6$) : δ 10.93 (1H, s), 10.87 (1H, s), 8.45 (8H, d, J = 3.6 Hz), 8.31 (1H, d, J = 7.6 Hz), 8.06 (1H, t), 7.68 (8H, t, J = 7.6 Hz), 7.50 (1H, d, J = 8.0 Hz), 7.42 (8H, d, J = 7.6 Hz), 7.20 (8H, dd, J = 1.6 Hz, 6.0 Hz), 6.98 (4H, d, J = 2.4 Hz), 6.73 (1H, t), 4.51-4.39 (2H, m), 3.73 (16H, s), 3.63 (8H, d, J = 2.8 Hz), 3.42 (3H, s), 3.15 (4H, m), 2.89-2.66 (6H, m), 2.31-2.07 (4H, m), 1.32 (9H, s). ESI-TOF-MS *m/z* $[M + H]^+$ calcd for $C_{85}H_{96}N_{16}O_{11}$ 1520.77; Found 1520.78.

Synthesis of **22**

To an ice-cooled solution of the crude **21** (43 mg) in CH_2Cl_2 (1 mL) was added dropwise TFA (1 mL) and the solution was stirred for 1 h at rt. After removal of the solvent in vacuo, the residue was dissolved in H_2O. The mixture was neutralized with aq. NH_3 on ice-cooling and extracted with CH_2Cl_2 (x2). The organic layer was washed with brine followed by drying over Na_2SO_4. The solution was concentrated by evaporation to give **22** (41 mg, quant) as a colorless oil. This material contained a small amount of impurities but was used for the next reaction without further purification.

^{1}H-NMR (400 MHz, $CDCl_3$) : δ 11.03 (2H, br), 8.49 (8H, t, J = 5.6 Hz), 7.58 (8H, dd, J = 8.0 Hz, 11.6 Hz), 7.42 (8H, t, J = 7.2 Hz), 7.37 (1H, t, J = 7.2 Hz), 7.12-7.08 (8H, m), 7.02 (2H, s), 6.98 (2H, s), 4.64-4-57 (2H, m), 3.83 (16H, s), 3.77-3.71 (8H, m), 3.56 (3H, s), 3.55-3.46 (6H, m), 3.10-2.82 (8H, m), 2.20-2.04 (4H, m). ESI-TOF-MS *m/z* $[M + H]^+$ calcd for $C_{80}H_{91}N_{16}O_9$ 1419.71; Found 1419.73.

Synthesis of **8**

To an ice-cooled solution of 4-(dimethylamino)-2-butenoic acid hydrochloride (3.4 mg, 21 μmol) in dry DMF (1 mL) was added DIEA (10 μL, 56 μmol), Py-BOP (11 mg, 21 μmol) and the crude **22** (20 mg). The solution was stirred for 14.5 h at rt. After dilution with AcOEt, the solution was washed with saturated $NaHCO_3$ and brine followed by drying over Na_2SO_4. After removal of the solvent by evaporation, the residue was purified by column chromatography on SiO_2 ($CHCl_3$: MeOH : NH_3 = 400 : 4 : 1→200 : 4 : 1→180 : 30 : 1) to give **8** (7 mg, 33%) as a colorless oil.

^{1}H-NMR (400 MHz, DMSO-d_6) : δ 10.93 (1H, s), 10.88 (1H, s), 8.46 (8H, d, J = 3.6 Hz), 8.46 (8H, d, J = 3.6 Hz), 8.36 (1H, m), 8.20 (1H, m), 8.02 (1H, m), 7.68 (8H, t, J = 7.2 Hz), 7.52 (1H, d), 7.41 (8H, d, J = 8.0 Hz), 7.20 (8H, d, J = 7.6 Hz), 6.98 (4H, s), 6.52 (1H, m), 6.06 (1H, m), 4.56-4.38 (4H, m), 3.72-3.63 (26H, m), 3.42 (3H, s), 3.15 (4H, m), 2.90 (6H, m), 2.30-2.11 (4H, m), 2.06 (6H, s). ESI-TOF-MS *m/z* $[M + H]^+$ calcd for $C_{86}H_{100}N_{17}O_{10}$ 1530.78; Found 1530.78.

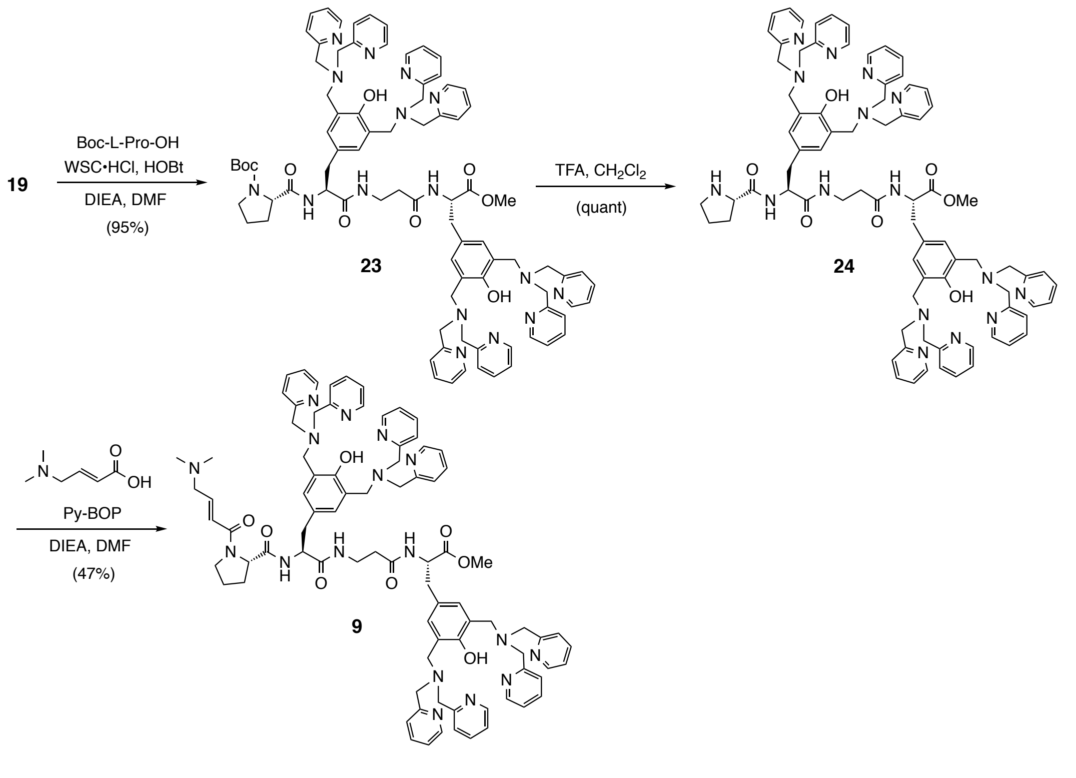

Synthesis of **23**

To an ice-cooled solution of **19** (20 mg, 16 μmol) in dry DMF (1.0 mL) was added WSCI•HCl (3.6 mg, 19 μmol), HOBt•H_2O (2.9 mg, 19 μmol), DIEA (12 μL, 75 μmol) and Boc-L-Pro (4 mg, 19 μmol). The solution was stirred for 13 h at rt. After dilution with AcOEt, the solution was washed with saturated $NaHCO_3$ and brine followed by drying over Na_2SO_4. After removal of the solvent by evaporation, the residue was purified by column chromatography on SiO_2 ($CHCl_3$: MeOH : NH_3 = 400 : 4 : 1→160 : 20 : 1) to give **23** (22 mg, 95%) as a colorless amorphous powder.

^{1}H-NMR (400 MHz, $CDCl_3$) : δ 10.98 (2H, br), 8.49 (8H, d, *J* = 4.0 Hz), 7.58 (8H, dd, *J* = 8.0 Hz, 11.6 Hz), 7.45 (8H, t, *J* = 6.8 Hz), 7.12-7.08 (8H, m), 6.99 (4H, s), 6.77 (2H, m), 4.68-4.53 (2H, m), 4.11 (1H, m), 3.83-3.75 (24H, m), 3.57 (3H, s), 3.20-2.96 (10H, m), 2.22 (4H, m), 1.62 (9H, br). ESI-TOF-MS *m/z* $[M + H]^+$ calcd for $C_{84}H_{95}N_{16}O_9$ 1472.75; Found 1472.75.

Synthesis of **24**

To an ice-cooled solution of **23** (20 mg, 13.6μmol) in CH_2Cl_2 (1 mL) was added dropwise TFA (1 mL) and the solution was stirred for 1 h at rt. After removal of the solvent in vacuo, the residue was dissolved in H_2O. The mixture was neutralized with aq. NH_3 on ice-cooling and extracted

with CH_2Cl_2. The organic layer was washed with brine followed by drying over Na_2SO_4. The solution was concentrated by evaporation to give **24** (24 mg, quant) as a colorless oil.

^{1}H-NMR (400 MHz, $CDCl_3$) : δ 10.95 (2H, br), 8.49 (8H, t, *J* = 3.2 Hz), 8.02 (1H, m), 7.58 (8H, dd, *J* = 7.2 Hz, 10.4 Hz), 7.45 (8H, t, *J* = 8.8 Hz), 7.37 (1H, t, *J* = 8.0 Hz), 7.10-7.02 (8H, m), 7.00 (5H, s), 4.63 (2H, dd, *J* = 5.2 Hz), 4.41 (2H, m), 4.11 (1H, m), 3.87-3.75 (24H, m), 3.60 (3H, s), 3.22-2.10(14H, m). ESI-TOF-MS *m/z* $[M + H]^+$ calcd for $C_{79}H_{87}N_{16}O_7$ 1371.69; Found 1371.70.

Synthesis of **9**

To an ice-cooled solution of 4-(dimethylamino)-2-butenoic acid hydrochloride (3.4 mg, 21 μmol) in dry DMF (1 mL) was added a mixture of DIEA (10 μL, 56 μmol), Py-BOP (11 mg, 21 μmol) and **24** (22 mg, 14 μmol). The solution was stirred for 18 h at rt. After dilution with AcOEt, the solution was washed with saturated $NaHCO_3$ and brine followed by drying over Na_2SO_4. After removal of the solvent by evaporation, the residue was purified by column chromatography on SiO_2 ($CHCl_3$: MeOH : NH_3 = 400 : 4 : 1→160 : 20 : 1) to give **9** (11 mg, 47%) as a colorless amorphous powder.

^{1}H-NMR (400 MHz, DMSO-d_6) : δ 10.94 (2H, d), 8.46 (8H, d), 8.30 (1H, d, *J* = 10 Hz), 7.79 (1H, m), 7.68 (8H, t, *J* = 7.6 Hz), 7.44 (8H, t, *J* = 8.0 Hz), 7.21 (8H, t, *J* = 5.6 Hz), 6.99 (4H, s), 6.58 (1H, m), 6.32 (1H, m), 4.41 (2H, m), 3.72 (16H, s), 3.64 (8H, s), 3.42 (3H, s), 3.18-2.78 (12H, m), 2.12 (6H, br), 1.87 (2H, m). ESI-TOF-MS *m/z* $[M + H]^+$ calcd for $C_{85}H_{96}N_{17}O_8$ 1482.76; Found 1482.76.

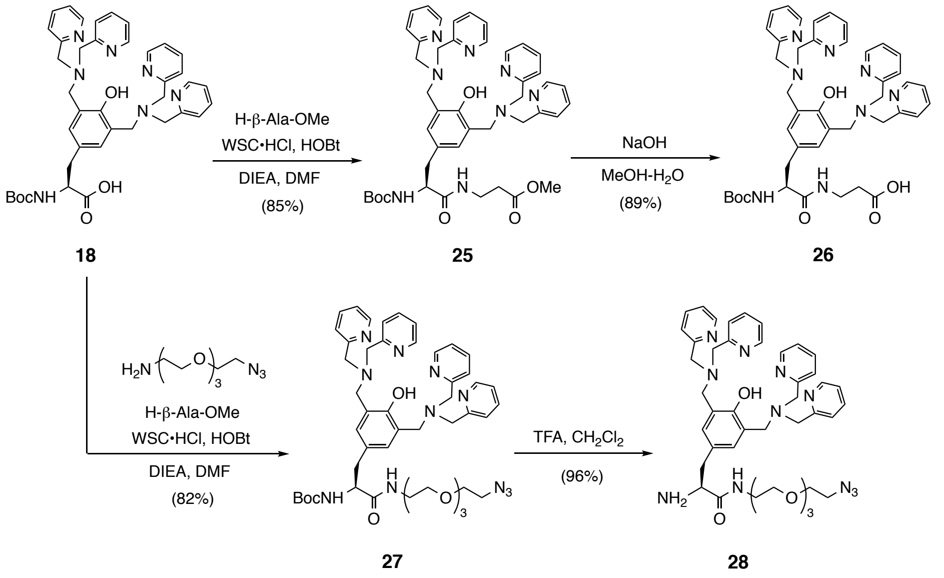

Synthesis of **25**

To an ice-cooled solution of **18** (1.0 g, 1.42 mmol) in dry DMF (6 mL) was added WSCI•HCl (355 mg, 1.85 mmol), HOBt•H_2O (283 mg, 1.85 mmol), DIEA (0.9 mL, 5.68 mmol) and H-□□la-OMe•HCl (258 mg, 1.85 mmol). The solution was stirred for 6 h at rt. After dilution with AcOEt, the solution was washed with saturated $NaHCO_3$ and brine followed by drying over Na_2SO_4. After removal of the solvent by evaporation, the residue was purified by column chromatography on SiO_2 ($CHCl_3$: MeOH : NH_3 = 300 : 10 : 1 → 200 : 10 : 1) to give **25** (953 mg, 85%) as a colorless amorphous powder.

^{1}H-NMR(400 MHz, $CDCl_3$): δ 10.98 (1H, br), 8.54 (4H, d, J = 4.8 Hz), 7.60 (4H, t, J = 6.8 Hz), 7.45 (4H, d, J = 8.0 Hz), 7.12 (4H, t, J = 8.0 Hz), 7.04 (2H, s), 5.32 (1H, m), 4.30 (1H, m), 3.90-3.70 (12H, m), 3.46 (3H, s), 3.31-3.23 (2H, m), 3.02-2.97 (1H, m), 2.87-2.81 (1H, m), 2.34-2.17 (2H, m), 1.36 (9H, s). ESI-TOF-MS m/z $[M + H]^+$ calcd for $C_{44}H_{53}N_8O_6$ 789.41; Found 789.41.

Synthesis of **26**

To an ice-cooled solution of **25** (916 mg, 1.16 mmol) in MeOH-H_2O (18 : 5 (v/v), 23 mL) was added 5 N NaOH (696 □L, 3.48 mmol) and the solution was stirred for 7 h at 0 °C. After dilution with water, the solution was washed with diethyl ether (x2). The pH was adjusted to 5 and extracted with $CHCl_3$ (x2). The organic phase was dried over Na_2SO_4 and concentrated in vacuo. The residue was washed with hexane-*i*-PrOH (1 : 1) to give **26** (804 mg, 89%) as a colorless amorphous powder.

^{1}H-NMR (400 MHz, $CDCl_3$) : δ 8.54 (4H, m), 7.70-7.52 (8H, m), 7.13 (4H, m), 7.01 (2H, s), 6.70 (1H, m), 5.55 (1H, m), 4.37 (1H, m), 4.00 (12H, m), 3.46 (1H, m), 3.26 (1H, m), 3.09-3.07 (1H,

m), 2.76-2.70 (1H, m), 2.22 (2H, m). ESI-TOF-MS *m/z* $[M + H]^+$ calcd for $C_{43}H_{51}N_8O_6$ 775.39; Found 775.38.

Synthesis of **27**

To an ice-cooled solution of **18** (200 mg, 0.28 mmol) in dry DMF (3.0 mL) was added WSCI•HCl (83 mg, 0.43 mmol), HOBt•H_2O (64 mg, 0.43 mmol), DIEA (148 μL, 0.85 mmol) and 11-azido-3,6,9-trioxaundecan-1-amine (56 μL, 0.28 mmol). The solution was stirred overnight at rt. After dilution with AcOEt, the solution was washed with saturated $NaHCO_3$ and brine followed by drying over Na_2SO_4. After removal of the solvent by evaporation, the residue was purified by column chromatography on SiO_2 ($CHCl_3$: MeOH : NH_3 = 400 : 10 : 1 → 300 : 10 : 1) to give **27** (211 mg, 82%) as a colorless oil.

^{1}H-NMR(400 MHz, $CDCl_3$): δ 10.97 (1H, br), 8.53 (4H, d, *J* = 3.6 Hz), 7.60 (4H, t, *J* = 7.6 Hz), 7.45 (4H, d, *J* = 8.0 Hz), 7.12 (4H, t, *J* = 7.6 Hz), 7.05 (2H, s), 6.90 (1H, br), 4.33 (1H, m), 3.85 (8H, s), 3.75 (4H, dd, *J* = 19.2 Hz, 14 Hz), 3.66-3.24 (19H, m), 3.00-2.86 (2H, m), 1.35 (9H, s). ESI-TOF-MS *m/z* $[M + H]^+$ calcd for $C_{48}H_{62}N_{11}O_7$ 904.48; Found 904.50.

Synthesis of **28**

To an ice-cooled solution of **27** (210 mg, 232 μmol) in CH_2Cl_2 (1.5 mL) was added dropwise TFA (1.5 mL) and the solution was stirred for 1 h at rt. After removal of the solvent in vacuo, the residue was dissolved in H_2O. The mixture was neutralized with aq. NH_3 on ice-cooling, diluted with saturated $NaHCO_3$, and then extracted with CH_2Cl_2 (x2). The organic layer was dried over Na_2SO_4. The solution was concentrated by evaporation to give **28** (176 mg, 96%) as a pale yellow oil.

^{1}H-NMR (400 MHz, $CDCl_3$) : δ 10.99 (1H, br), 8.51 (4H, d, *J* = 4.8 Hz), 7.63-7.59 (4H, m), 7.47 (4H, d, *J* = 8.0 Hz), 7.11 (4H, t, *J* = 6.4 Hz), 7.05 (2H, s), 3.86 (8H, s), 3.80 (4H, s), 3.69-3.34 (16H, m), 3.18-3.14 (2H, m), 2.58-2.51 (1H, m). ESI-TOF-MS *m/z* $[M + H]^+$ calcd for $C_{43}H_{54}N_{11}O_5$ 804.43; Found 804.44.

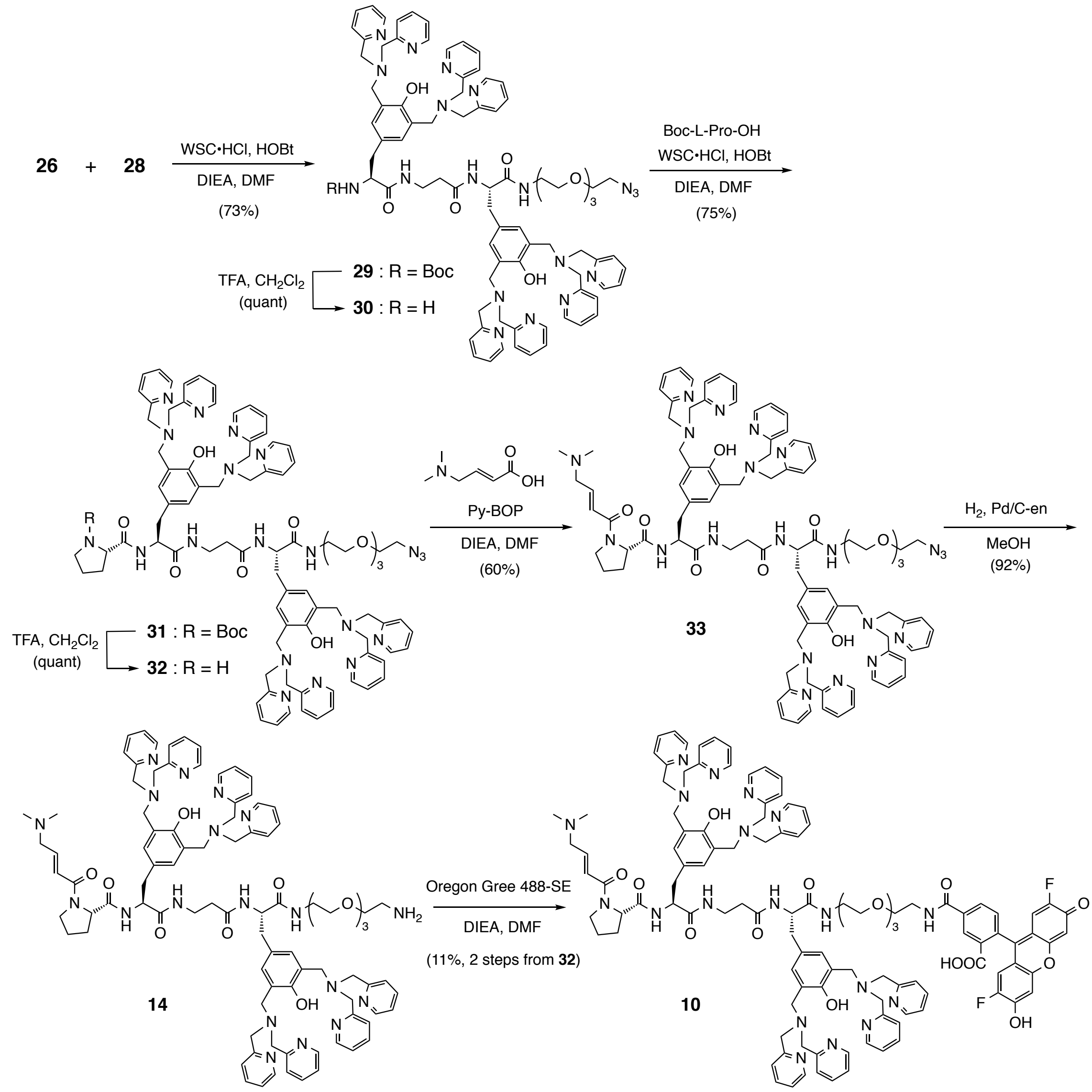

Synthesis of **29**

To a solution of **28** (99 mg, 132 μmol) in dry DMF (2 mL) was added WSCI•HCl (36 mg, 185 μmol), HOBt•H_2O (28 mg, 185 μmol), DIEA (86 μL, 492 μmol) and **26** (115 mg, 148 μmol). The solution was stirred overnight at rt. After dilution with AcOEt, the solution was washed with saturated $NaHCO_3$ and brine followed by drying over Na_2SO_4. After removal of the solvent by evaporation, the residue was purified by column chromatography on SiO_2 ($CHCl_3$: MeOH : NH_3 = 200 : 10 : 1) to give **29** (140 mg, 73%) as a colorless amorphous powder.

^{1}H-NMR (400 MHz, $CDCl_3$) : δ 10.99 (2H, br), 8.54-8.51 (8H, m), 7.61-7.58 (8H, m), 7.52-7.43 (8H, m), 7.14-7.02 (12H, m), 5.44 (1H, m), 4.62-4.60 (1H, m), 4.29 (1H, m), 3.84-3.18 (42H, m),

2.95-2.85 (6H, m), 2.27-2.17 (2H, m), 1.27 (9H, s). ESI-TOF-MS 1561.78 [M + H]$^+$. ESI-TOF-MS *m/z* [M + H]$^+$ calcd for $C_{86}H_{102}N_{19}O_{10}$ 1561.88; Found 1561.78.

Synthesis of **30**

To an ice-cooled solution of **29** (139 mg, 89 μmol) in CH_2Cl_2 (2 mL) was added dropwise TFA (2 mL) and the solution was stirred for 2 h at rt. After removal of the solvent in vacuo, the residue was dissolved in H_2O. The mixture was neutralized with aq. NH_3 on ice-cooling, diluted with saturated $NaHCO_3$, and then extracted with CH_2Cl_2. The organic layer was dried over Na_2SO_4. The solution was concentrated by evaporation to give **30** (130 mg, quant) as a pale red amorphous powder.

^{1}H-NMR (400 MHz, $CDCl_3$) : δ 10.99 (2H, br), 8.50 (8H, dd, *J* = 7.6 Hz, 4.0 Hz), 7.62-7.57 (8H, m), 7.48-7.43 (8H, t, *J* = 8.8 Hz), 7.14-7.02 (12H, m), 4.66 (1H, m), 3.84-3.06 (46H, m), 2.96-2.86 (2H, m), 2.31 (2H, m). ESI-TOF-MS *m/z* [M + H]$^+$ calcd for $C_{81}H_{94}N_{19}O_8$ 1460.75; Found 1460.76.

Synthesis of **31**

To an ice-cooled solution of **30** (40 mg, 27 μmol) in dry DMF (1.5 mL) was added WSCI•HCl (6.4 mg, 33 μmol), HOBt•H_2O (5 mg, 25 μmol), DIEA (22 μL, 132 μmol) and Boc-L-Pro (7 mg, 33 μmol). The solution was stirred for 4.5 h at rt. After dilution with AcOEt, the solution was washed with saturated $NaHCO_3$ and brine followed by drying over Na_2SO_4. After removal of the solvent by evaporation, the residue was purified by column chromatography on SiO_2 ($CHCl_3$: MeOH : NH_3 = 400 : 4 : 1 → 160 : 20 : 1) to give **31** (34 mg, 75%) as a pale yellow oil.

^{1}H-NMR (400 MHz, $CDCl_3$) : δ 10.95-10.94 (2H, m), 8.52 (8H, t, *J* = 4.0 Hz), 7.58 (8H, t, *J* = 7.6 Hz), 7.44 (8H, t, *J* = 6.8 Hz), 7.32 (1H, m), 7.13-7.11 (8H, t, *J* = 5.2 Hz), 7.02-7.00 (3H, m), 6.72 (1H, m), 4.60-4.52 (2H, m), 4.11 (1H, m), 3.83 (16H, s), 3.71-3.18 (28H, m), 2.90-2.89 (3H, m), 2.25 (1H, m), 1.93 (2H, m), 1.71 (13H, m). ESI-TOF-MS *m/z* [M + H]$^+$ calcd for $C_{91}H_{109}N_{20}O_{11}$ 1658.86; Found 1658.89.

Synthesis of **32**

To an ice-cooled solution of **31** (32 mg, 19 μmol) in dry CH_2Cl_2 (1.0 mL) was added dropwise TFA (1.0 mL) and the solution was stirred for 1 h at rt. After removal of the solvent in vacuo, the residue was dissolved in H_2O. The mixture was neutralized with aq. NH_3 on ice-cooling, diluted with saturated $NaHCO_3$, and then extracted with CH_2Cl_2 (x2). The organic layer was washed with

brine and dried over Na_2SO_4. The solution was concentrated by evaporation to give **32** (32 mg, quant) as a colorless oil.

^{1}H-NMR (400 MHz, $CDCl_3$) : δ 10.95 (2H, brs), 8.51 (8H, t, J = 5.2 Hz), 8.04 (1H, m), 7.58 (8H, t, J = 8.0 Hz), 7.43 (8H, t, J = 8.0 Hz), 7.14-7.10 (8H, m), 7.04 (4H, s), 4.57-4.47 (2H, m), 3.83 (16H, s), 3.80-2.52 (31H, m), 2.27-1.66(7H, m). ESI-TOF-MS m/z $[M + H]^+$ calcd for $C_{86}H_{101}N_{20}O_9$ 1558.81; Found 1558.84.

Synthesis of **33**

To an ice-cooled solution of 4-(dimethylamino)-2-butenoic acid hydrochloride (7.0 mg, 43 μmol) in dry DMF (1 mL) was added DIEA (19 μL, 116 μmol), Py-BOP (22 mg, 43 μmol) and **32** (30 mg, 19 μmol). The solution was stirred overnight at rt. After dilution with water, the solution was washed with saturated $NaHCO_3$ and brine followed by drying over Na_2SO_4. After removal of the solvent by evaporation, the residue was purified by column chromatography on SiO_2 ($CHCl_3$: MeOH : NH_3 = 400 : 4 : 1→160 : 20 : 1) to give **33** (19 mg, 60%) as a colorless oil.

^{1}H-NMR (400 MHz, $CDCl_3$) : δ 10.95 (2H, br), 8.50 (8H, dd, J = 11.2 Hz, 4.4 Hz), 7.58 (8H, t, J = 7.6 Hz), 7.43 (8H, t, J = 8.4 Hz), 7.34-7.32 (2H, m), 7.13-7.09 (8H, m), 7.01 (4H, d, J = 5.2 Hz), 6.80-6.74 (1H, m), 4.60-4.58 (1H, m), 4.38 (2H, m), 3.83 (16H, s), 3.79-3.01 (28H, m), 2.88-2.84 (2H, m), 2.39-2.35 (1H, m), 2.21 (8H, br), 1.75 (4H, m). ESI-TOF-MS m/z $[M + H]^+$ calcd for $C_{92}H_{110}N_{21}O_{10}$ 1669.88; Found 1669.91.

Synthesis of **14**

A solution of **33** (18 mg, 11μmol) and Pd/C-ethylenediamine complex (10 mg) in MeOH-CH_2Cl_2 (1 : 1, 5 mL) was vigorously stirred for 5 h under H_2 atmosphere at rt. After filtration, the solvent was removed by evaporation to give the crude **14** (20 mg) as a pale yellow oil. This material used for the next reaction without further purification.

ESI-TOF-MS m/z $[M + H]^+$ calcd for $C_{92}H_{112}N_{19}O_{10}$ 1642.88; Found 1642.91.

Synthesis of **10**

A mixture of crude **14** (10 mg), DIEA (18 μL, 108 μmol), Oregon Green 488-SE (2.5 mg, 5 μmol) in dry DMF (2 mL) was stirred for 3 h at rt. The reaction was monitored by reverse-phase HPLC. Purification by reverse-phase HPLC and subsequent lyophilization gave **10** (2.4 mg, 11%

in two steps) as a yellow powder. The purity of **10** was determined to be > 95% by HPLC analysis (UV, λ = 220 nm).

HPLC conditions: column; YMC-pack ODS-A, 250 x 20 mm, mobile phase; CH_3CN (containing 0.1% TFA) / H_2O (containing 0.1% TFA) = 5 / 95 (10 min) → 50 / 50 (linear gradient over 50 min), flow rate; 9.9 mL/min, detection; UV (λ = 220 nm).

ESI-TOF-MS *m/z* $[M + H]^+$ calcd for $C_{113}H_{120}F_2N_{19}O_{16}$ 2037.92; Found 2037.91.

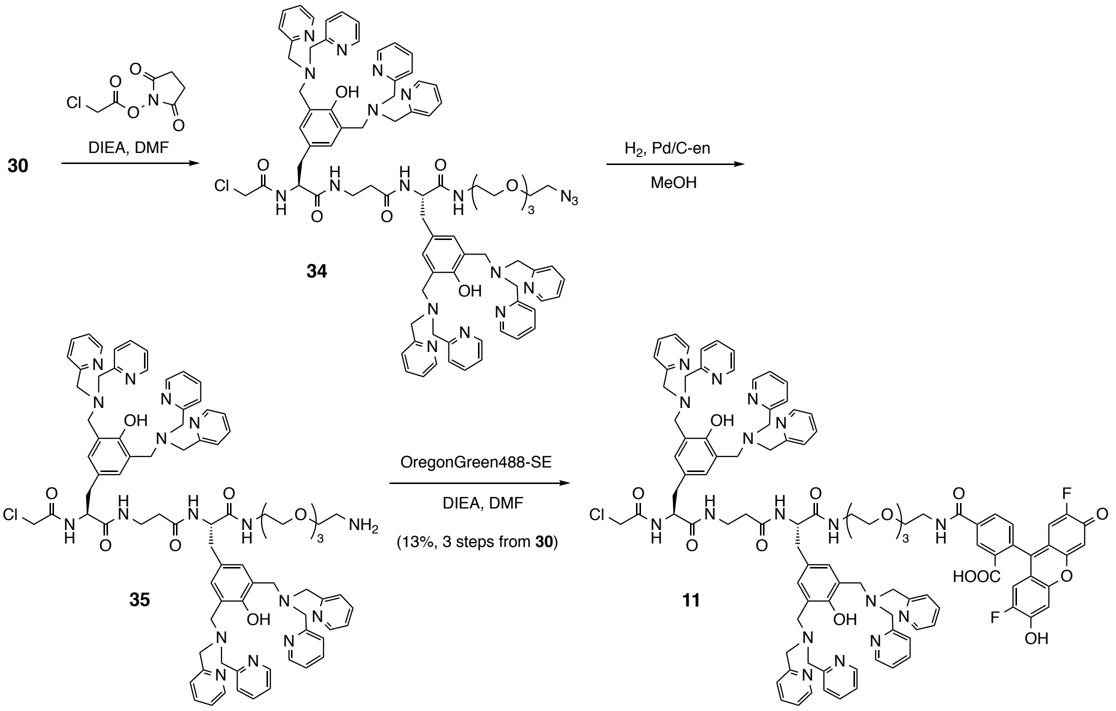

Synthesis of **34**

To a solution of **30** (20 mg, 14 μmol) in dry DMF (1.0 mL) was added DIEA (7 μL, 41 μmol) and chloroacetic acid *N*-hydroxysuccinimidyl ester (4.0 mg, 19 μmol) and the solution was stirred for 2 h at rt. After dilution with AcOEt, the solution was washed with saturated $NaHCO_3$ and brine followed by drying over Na_2SO_4. The solvent was removed by evaporation to give crude **34** (21 mg) as a pale yellow oil. This material used for the next reaction without further purification.

ESI-TOF-MS *m/z* $[M + H]^+$ calcd for $C_{83}H_{95}ClN_{19}O_9$ 1536.72; Found 1536.72.

Synthesis of **35**

A solution of the crude **34** (21 mg) and Pd/C-ethylenediamine complex (10 mg) in MeOH-CH_2Cl_2 (1 : 1, 5 mL) was vigorously stirred for 5 h under H_2 atmosphere at rt. After filtration, the solvent was removed by evaporation to give crude **35** (17 mg) as a pale yellow oil. This material used for the next reaction without further purification.

ESI-TOF-MS *m/z* $[M + H]^+$ calcd for $C_{83}H_{97}ClN_{17}O_9$ 1510.73; Found 1510.74.

Synthesis of **11**

A mixture of the crude **35** (17 mg), DIEA (18 μL, 108 μmol), Oregon Green 488-SE (2.5 mg, 5 μmol) in dry DMF (1.5 mL) was stirred for 3 h at rt. The reaction was monitored by reverse-phase HPLC. Purification by reverse-phase HPLC and lyophilization gave **11** (3.3 mg, 13% in three steps) as a yellow powder. The purity of **11** was determined to be > 95% by HPLC analysis (UV, λ = 220 nm).

HPLC conditions: column; YMC-pack ODS-A, 250 x 20 mm, mobile phase; CH_3CN (containing 0.1% TFA) / H_2O (containing 0.1% TFA) = 0 / 100 → 50 / 50 (linear gradient over 50 min), flow rate; 9.9 mL/min, detection; UV (λ = 220 nm).

ESI-TOF-MS *m/z* $[M + H]^+$ calcd for $C_{104}H_{105}ClF_2N_{17}O_{15}$ 1905.77; Found 1905.76.

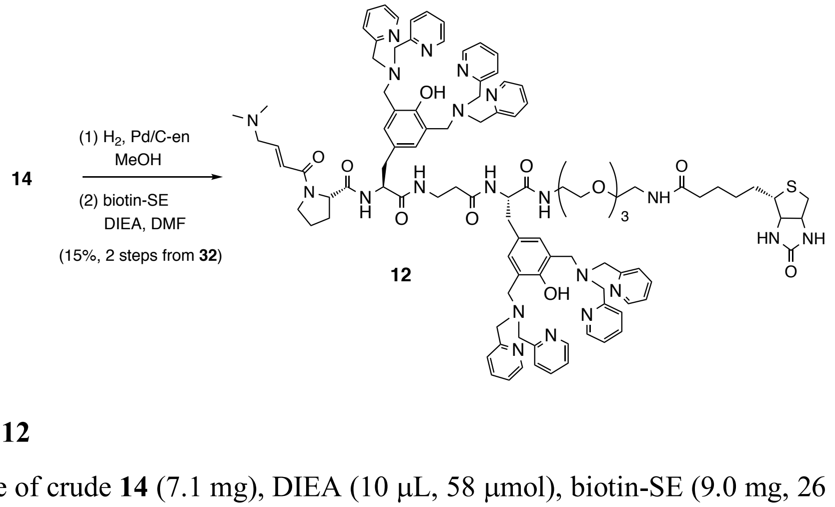

Synthesis of **12**

A mixture of crude **14** (7.1 mg), DIEA (10 μL, 58 μmol), biotin-SE (9.0 mg, 26 μmol) in dry DMF (1 mL) was stirred for 13 h at rt. The reaction was monitored by reverse-phase HPLC. Purification by reverse-phase HPLC and lyophilization gave **12** (2.0 mg, 15% in two steps) as a colorless powder. The purity of **12** was determined to be > 95% by HPLC analysis (UV, λ = 220 nm).

HPLC conditions: column; YMC-pack ODS-A, 250 x 10 mm, mobile phase; CH_3CN (containing 0.1% TFA) / H_2O (containing 0.1% TFA) = 5 / 95 (10 min) → 45 / 55 (linear gradient over 40 min), flow rate; 3.0 mL/min, detection; UV (λ = 220 nm).

ESI-TOF-MS *m/z* $[M + H]^{3+}$ calcd for $C_{102}H_{126}N_{21}O_{12}S$ 623.32; Found 624.33.

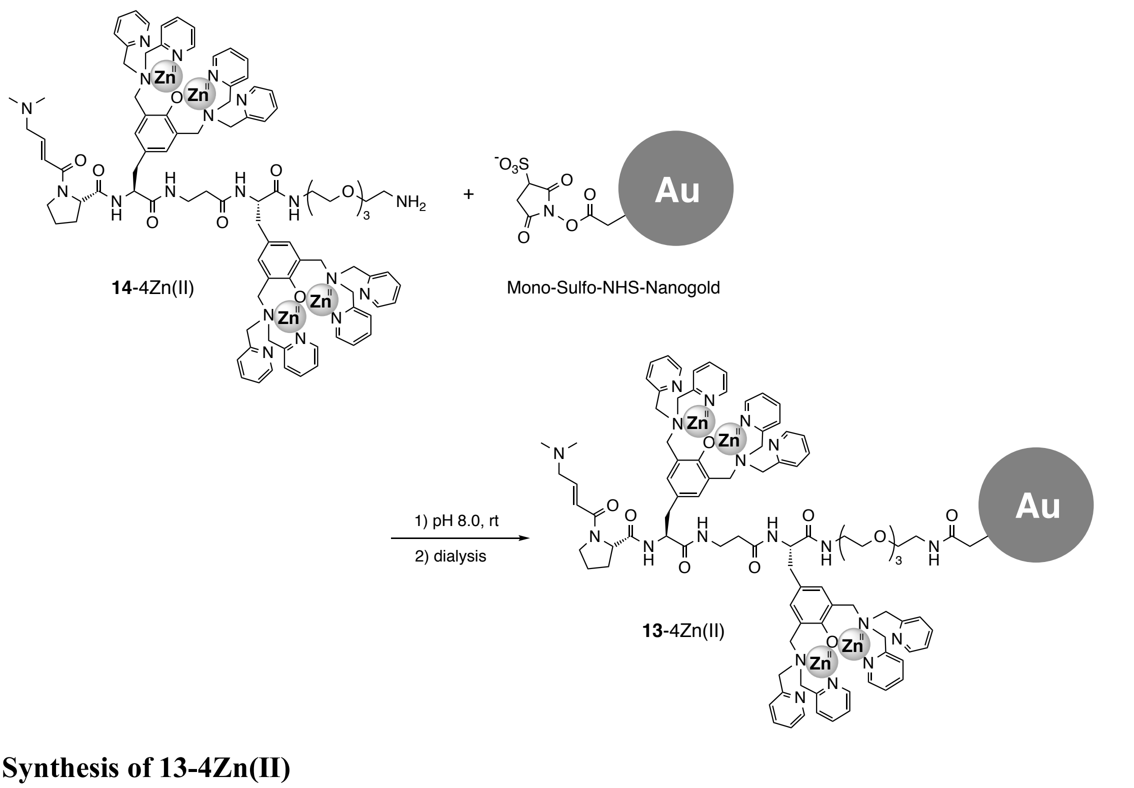

Synthesis of 13-4Zn(II)

Mono-Sulfo-NHS-Nanogold (purchased from Nanoprobes) (6 nmol) was dissolved in MilliQ water (200 μL) and mixed with the solution (200 μL) of the zinc complex **14**-4Zn(II) (~ 120 mmol) in 50 mM HEPES (pH 8.0), in which concentration of **14**-4Zn(II) was determined by UV absorbance at 280 nm using the measured extinction coefficient ($\varepsilon_{280\ nm}$ = 5170 $cm^{-1}M^{-1}$). The mixture was incubated at rt for 3 h with gentle shaking. The mixture was transferred into a dialysis membrane (MWCO: 14,000) and dialyzed (1 h x 3) against 300 mL of 50 mM HEPES (pH 7.2) containing 50 μM $ZnCl_2$ at rt to remove the excess of **14**-4Zn(II). The conjugation of **14**-4Zn(II) with the nanogold was confirmed by UV absorbance measurement at 280 nm and 420 nm, in which the absorbance ratio ($A_{280\ nm}$ / $A_{420\ nm}$ = 2.77) of the nanogold and the extinction coefficient of **14**-4Zn(II) were used to estimate the labeling yield.

References

Yamamori S, Itakura M, Sugaya D, Katsumata O, Sakagami H, and Takahashi M. (2011). Differential expression of SNAP-25 family proteins in the mouse brain. *J. Comp. Neurol. 519*, 916-932.

Luján, R. *et al.* (2018). Differential association of $GABA_B$ receptors with their effector ion channels in Purkinje cells. *Brain Struct. Func. 223*, 1565-1587.

Miki, T., Kaufmann, .W. A., Malagon, G., Gomez, L., Tabuchi, K., Watanabe M., Shigemoto, R., and Marty, A. (2017). Numbers of presynaptic Ca^{2+} channel clusters match those of functionally defined vesicular docking sites in single central synapses. *Proceed. Natl. Acad. Sci. USA 114*, E5246-E5255.

Nonaka, H., Fujishima, S., Uchinomiya, S., Ojida, A., and Hamachi, I. (2010). Selective covalent labeling of Tag-fused GPCR proteins on live cell surface with a synthetic probe for their functional analysis. *J. Am. Chem. Soc.*, *132*, 9301-9309.
